# EBI2-oxysterol signalling regulates VE-cadherin expression and multiple sclerosis CD4^+^ T cell attachment to a human tri-cell spheroid blood-brain barrier model

**DOI:** 10.1016/j.bbih.2025.101045

**Published:** 2025-06-20

**Authors:** Fionä Caratis, Inez Mruk, Klaudia Konieczna-Wolska, Bartłomiej Rojek, Marek Hałas, Paulina Czaplewska, Bartosz Karaszewski, Tomomi Furihata, Aleksandra Rutkowska

**Affiliations:** a Department of Anatomy and Neurobiology, Medical University of Gdańsk, Gdańsk, Poland; b Laboratory of Mass Spectrometry-Core Facility Laboratories, Intercollegiate Faculty of Biotechnology UG and MUG, University of Gdańsk, Gdańsk, Poland; c Tri-City Central Animal Laboratory Research and Service Center, Medical University of Gdańsk, Gdańsk, Poland; d Department of Adult Neurology, Medical University of Gdańsk and University Clinical Center, Gdańsk, Poland; e Brain Diseases Centre, Medical University of Gdańsk, Gdańsk, Poland; f Laboratory of Clinical Pharmacy and Experimental Therapeutics, School of Pharmacy, Tokyo University of Pharmacy and Life Sciences, Hachioji, Tokyo, Japan

**Keywords:** Multiple sclerosis, Neuroinflammation, Oxysterols, EBI2, Blood-brain barrier, Cerebrospinal fluid, CD4^+^ T cells, VE-Cadherin

## Abstract

Changes in the function of the blood-brain barrier (BBB) are one of the hallmarks of multiple sclerosis (MS) and are observed at very early stages of the disease. Several disease-modifying therapies for MS regulate tight junction and adherence junction proteins in the BBB thus limiting the entry of peripheral immune cells into the central nervous system (CNS). The Epstein-Barr virus-induced gene 2 (EBI2) was shown to drive immune cell migration towards high concentration of its endogenous ligand, oxysterol 7α,25OHC, which concentrations increase during inflammation in the CNS. Here, the data showed upregulated transcripts of *EBI2* and *CH25H*, the first enzyme in 7α, 25OHC synthesis pathway, in MS brain lesions. *In vitro*, cerebrospinal fluid (CSF) from patients with MS downregulated *HSD3B7*, the 7α, 25OHC degrading enzyme, and *VE-cadherin* levels in the tri-cell human BBB spheroid model. Importantly, EBI2 signalling mediated the attachment of MS patient-derived CD4^+^ T cells to the BBB spheroids. The data raises the possibility that elevated oxysterol levels in an inflamed brain might trigger a downregulation of VE-cadherin in endothelial cells, potentially easing the CNS infiltration of EBI2-expressing immune cells. This process can be modulated through the use of EBI2 ligands, suggesting a potential pathway for therapeutic intervention.

## 1 Introduction

In multiple sclerosis (MS), a chronic autoinflammatory disease of the central nervous system (CNS), peripherally activated lymphocytes enter the brain parenchyma where they propagate inflammation, activate resident cells and damage myelin sheaths (Compston and Coles, 2002). In non-diseased CNS, the entry of immune cells is limited by the blood-brain barrier (BBB) but in MS, the integrity of the BBB is broken allowing the entrance of peripheral immune cells (Mirshafiey et al., 2014; Yang et al., 2007). The disruption of the BBB occurs at very early stages and can be detected in normal-appearing white matter (WM) before other MS symptoms (Cramer et al., 2015; Spencer et al., 2018). Acute lesions are typically centred around blood vessels where perivascular inflammation and immune cell infiltration is evident and changes in the expression and organisation of tight junction (TJs) proteins is observed mainly in active lesions (Spencer et al., 2018).

Animal studies have also highlighted the importance of an intact BBB in disease progression. In the cuprizone model, BBB permeability precedes demyelination, while in the experimental autoimmune encephalomyelitis (EAE) and Theiler's murine encephalomyelitis virus (TMEV) models, properly functioning BBB limited disease progression (Bennett et al., 2010; Berghoff et al., 2017; Olsen et al., 2007; Richard S Beard et al., 2025). The significance of disrupted BBB function in the pathogenesis of MS is also supported by the fact that the majority of licenced disease-modifying therapies (DMTs) for MS modulate the BBB to a greater or lesser extent (Correale et al., 2021). For instance, fingolimod upregulates TJ claudin-5 and downregulates adhesion protein vascular cell adhesion molecule 1 (VCAM1) (Annunziata et al., 2018; Foster et al., 2009). Natalizumab blocks alpha-4 integrin on lymphocytes and monocytes and thus inhibits their passage to the CNS (Engelhardt and Kappos, 2008).

The Epstein-Barr virus-induced gene 2 (EBI2, GPR183), one of the key immune system modulators, has been implicated in several chronic inflammatory and autoimmune diseases including rheumatoid arthritis, type 1 diabetes, inflammatory bowel disease and MS (Crick et al., 2017; Gatto et al., 2011; Klejbor et al., 2021; Konieczna-Wolska et al., 2024; Kutryb-Zajac et al., 2022; Pereira et al., 2009; Velasco-Estevez et al., 2021; Vigne et al., 2017; Wanke et al., 2017). Its expression is upregulated in memory CD4^+^ and CD8^+^ T cell subsets in natalizumab, but not dimethyl fumarate-treated patients with MS (Clottu et al., 2017). These patients’ cells also display increased migration towards the EBI2 endogenous ligand, oxysterol 7α,25-dihydroxycholesterol (7α,25OHC) *in vitro*, an effect that is inhibited with the EBI2 antagonist, NIBR189(Clottu et al., 2017). Raised levels of oxysterol 25OHC, the precursor to 7α,25OHC, were found in the brains of LPS-treated mice and in the cerebrospinal fluid (CSF) of patients with inflammatory CNS disease and reduced in the plasma of relapsing-remitting MS (RRMS) patients (Crick et al., 2017; Romero et al., 2024). Importantly, EBI2 is highly expressed in infiltrating immune and glial cells in MS lesions (Klejbor et al., 2021; Wanke et al., 2017)

The ligand, 7α,25OHC, is synthesized *in vivo* from cholesterol by the sequential enzymatic activity of cholesterol 25-hydroxylase (CH25H) and 25-hydroxycholesterol 7-alpha-hydroxylase (CYP7B1) and metabolized by 3 beta-hydroxy-Delta (5)-C27-steroid oxidoreductase (HSD3B7)(Russell, 2003). In EAE, the concentration of 7α, 25OHC in the mouse CNS was increased as a result of CH25H upregulation by microglia and CYP7B1 by infiltrating lymphocytes, while HSD3B7 was reduced (Wanke et al., 2017). Increased concentration of 7α, 25OHC enhance the migration of autoreactive T cells into the CNS during the early phases of transfer EAE. Importantly, the study found reduced numbers of CD4^+^ T cells in the CNS of EAE mice which received EBI2 deficient Th17 cells demonstrating that modifications of EBI2/7α, 25OHC signalling reduces CNS infiltration by the most encephalitogenic cell type in MS. Several studies have shown that downregulating the synthesis of 7α,25OHC, via CH25H or CYP7B1 enzymes, negatively regulates the immune system and attenuates CNS inflammation and the course of EAE (Bauman et al., 2009; Chalmin et al., 2015; Ruiz et al., 2023; Song et al., 2024).

EBI2 directs cell migration via 7α, 25OHC enabling appropriate immune responses (Clottu et al., 2017; Hannedouche et al., 2011; Liu et al., 2011; Preuss et al., 2014; Rutkowska et al., 2015, 2016; Yi et al., 2012). For instance, to ensure a correct positioning of activated B cells and dendritic cells-stimulated T cells in germinal centers, a tightly controlled 7α, 25OHC gradient is formed and maintained in the B cell follicles (Yi et al., 2012). Since B cells do not themselves express the 7α, 25OHC synthesizing (CH25H and CYP7B1) and degrading (HSD3B7) enzymes, they depend on other cells to regulate the gradient. Indeed, it has been shown that lymphoid stromal cells upregulate the levels of 7α, 25OHC via CH25H and CYP7B1 enzymes and follicular dendritic cells downregulate its levels via the HSD3B7 enzyme (Yi et al., 2012). Disruption of the gradient in the lymphoid tissue has similar consequences to receptor deficiency or its upregulation. In CH25H deficient mice, activated B cells fail to appropriately position themselves within the B cell follicles resulting in a three-fold decrease in the extent of antibody responses (Hannedouche et al., 2011). Conversely, saturating the lymphoid microenvironment with 7α, 25OHC or downregulation of CYP7B1 results in equal distribution of B cells in the lymphoid tissue and disrupted immune responses (Hannedouche et al., 2011; Liu et al., 2011)

We here investigated if the EBI2/oxysterol system is modulated in the BBB forming cells, that is endothelial cells, pericytes and astrocytes in the context of MS. To mimic the neuroinflammatory microenvironment in MS, we treated the human BBB cells and spheroids with CSF collected from patients with MS during first episode (acute MS). We also examined if the BBB can be modulated by the EBI2/oxysterol pathway during inflammation. Finally, chemotaxis and attachment of CD4^+^ T cells collected from the same patients with MS to the human tri-cell BBB spheroids was investigated.

## 2 Results

### 2.1 *EBI2* and *CH25H* are upregulated in MS brains

**Fig. 1:**
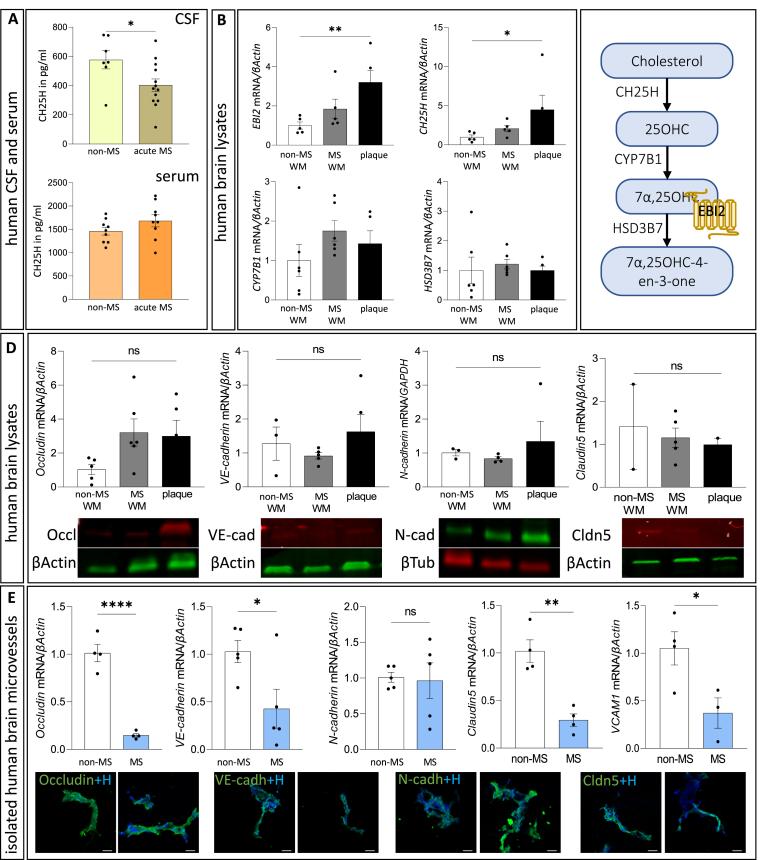
**EBI2 and CH25H are upregulated in MS brains.** A. CH25H quantification in the CSF with ELISA (top graph) showed a 30 % decrease in acute MS compared to non-MS group (402.8 ± 42.96 vs. 576.2 ± 61.85). There was no difference in serum CH25H levels between acute MS and non-MS groups (bottom graph). Data presented as mean ± SEM, unpaired *t*-test, ∗p < 0.05, CSF: N = 13 acute MS, N = 7 non-MS; serum: N = 9 acute MS, N = 9 non-MS. B. *EBI2* and *CH25H* were significantly upregulated in MS plaques compared to non-MS WM (respectively 3.2 ± 0.6 and 4.49 ± 1.85). Data presented as mean ± SEM, normalised to non-MS WM, Kruskal-Wallis and Dunn's multiple comparisons tests, ∗p < 0.05, ∗∗p < 0.01, N = 5 and 6 brain samples. C. Schematic representation of the oxysterol 7α, 25OHC synthesis pathway. D. There were no differences in the levels of *Occludin*, *VE-cadherin*, *N-cadherin* and *Claudin5* in the human brain lysates of non-MS WM, MS WM and plaque, both at mRNA and protein levels. One-way ANOVA, p > 0.05, N = 5 and 6 brain samples. Occludin ∼65 kDa, VE-cadherin ∼120 kDa, N-cadherin ∼130 kDa, Cldn5 ∼22 kDa, βActin ∼42 kDa, βTub 50–55 kDa E. The mRNA levels of *Occludin*, *VE-cadherin*, *Claudin5* and *VCAM1* were significantly upregulated in the isolated human brain microvessels. There were no differences in the levels of *N-cadherin*. Unpaired *t*-test, ∗p < 0.05, ∗∗∗∗p < 0.0001, N = 4 and 5 brains. Occludin, VE-cadherin, N-cadherin, and claudin-5 (all in green) are visualised in isolated human brain microvessels, as shown below the graphs. Scale 50 μm. (For interpretation of the references to colour in this figure legend, the reader is referred to the Web version of this article.)

To characterise the patient CSF samples before using them in *in vitro* experiments, we analysed their protein content with mass spectrometry (Additional Fig. 1). A total of 172 proteins were detected in the samples and 84 of these proteins were able to differentiate the two groups, the acute MS from non-MS group. Among these 84 proteins, 30 were significantly upregulated and 43 were downregulated in the acute MS group (Additional Table 1). No statistically meaningful differences between the sexes were observed. The deregulated proteins were allocated to the following subgroups based on function: myelin and oligodendrocytes, immune system regulation and inflammation, brain function, cholesterol and lipid metabolism, and other (Additional Fig. 1A). Notably, KEGG and Reactome pathway analysis using the STRING database identified five pathways related to cholesterol metabolism and 26 pathways involved in the innate immune system in the acute MS group (Additional Fig. 1B**)**. This brief proteomic analysis of the CSF confirmed the appropriateness of group allocation and diagnosis, supporting further *in vitro* studies with the CSF. Accordingly, we proceeded to examin the levels of CH25H, the first enzyme in the 7α,25-OHC synthesis pathway using the ELISA assay in the CSF and serum. Type 1 interferons, used as a treatment for MS, were shown to induce CH25H, increasing the synthesis of oxysterol 25OHC, the precursor to the EBI2 agonist, 7α, 25OHC (Forwell et al., 2016; Liu et al., 2013; Park and Scott, 2010). Here, the analysis of CH25H levels revealed reduced levels of CH25H in the CSF of patients with MS compared to non-MS controls and no differences in the serum (Fig. 1A). Further analysis revealed expression of *EBI2* and all three enzymes in the oxysterol pathway (*CH25H*, *CYP7B1* and *HSD3B7*) in human brain homogenates (Additional Fig. 2A) and isolated human brain microvessels (Additional Fig. 2B and C). We previously reported increased EBI2 presence specifically in astrocytes and microglia inside MS plaques (Klejbor et al., 2021) and here we report upregulated mRNA expression of *EBI2* and *CH25H* in MS plaques compared to non-MS WM (Fig. 1B and C). The levels of the second enzyme in 7α,25-OHC synthesis pathway, *CYP7B1*, and the degrading enzyme, *HSD3B7*, were at similar levels in the plaque and normal appearing WM in MS and non-MS controls. Further, in the same brain lysates, there were no significant differences in the protein and mRNA levels of *Occludin*, *vascular endothelial cadherin (VE)-cadherin*, *neural cadherin* (*N)-cadherin* and *Claudin5* (Fig. 1D and Additional Fig. 2D). However, *Occludin*, *VE-cadherin*, *Claudin5* and *VCAM1* were significantly downregulated in the microvessels isolated from MS brains compared to non-MS brains indicating disruption of the BBB in MS brains (Fig. 1E and Additional Fig. 2E).

### 2.2 CSF from patients with MS modulates the EBI2/oxysterol pathway in BBB cells

**Fig. 2:**
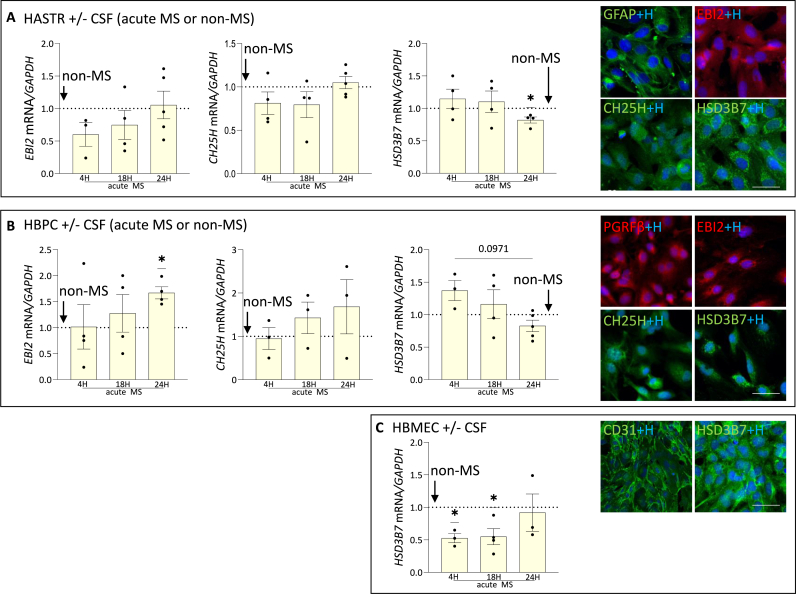
**CSF from acute patients with MS modulates the EBI2/oxysterol pathway in the BBB cells. A. CSF from patients with MS downregulated *HSD3B7*** transcripts after 24H (0.82 ± 0.05) while *EBI2 and CH25H were only insignificantly downregulated at earlier timepoints. Representative images of HASTR immunostained with anti-GFAP (astrocyte marker), anti-EBI2, anti-CH25H and anti-HSD3B7 antibodies.* Scale 50 μm B. In HBPCs, CSF from patients with MS upregulated *EBI2* expression (1.67 ± 0.11) after 24H. The expression of *CH25H displayed an increasing trend, while HSD3B7* was downregulated (0.83 ± 0.09, p < 0.1) after 24H. Representative images of HBPCs immunostained with anti-PDGFRβ (pericyte marker), anti-EBI2, anti-CH25H and anti-HSD3B7 antibodies. Scale 50 μm C. Finally, in HBMECs, *HSD3B7* expression was significantly downregulated by CSF from patients with MS at 4H (0.53 ± 0.07) and 18H (0.55 ± 0.12). Representative images of HBMECs immunostained with anti-CD31 (endothelial cell marker) and anti-HSD3B7 antibodies. Scale 50 μm. Dotted lines indicate treatment with non-MS CSF to which the data at each time point was normalised. Data presented as mean ± SEM, Wilcoxon or one-sample *t*-test, ∗p < 0.05, ∗∗p < 0.01, N = 3–5 independent experiments, dot represents the average of duplicates within each independent experiment.

Disruption of the BBB is one of the early hallmarks of MS and may indicate MS-associated neuroinflammation (Cramer et al., 2015; Spencer et al., 2018). To investigate if the EBI2/oxysterol system is modulated in the BBB forming cells, that is endothelial cells (HBMEC), pericytes (HBPC) and astrocytes (HASTR) in the context of MS we treated the cells *in vitro* with CSF collected from patients with MS during first episode (“acute MS”). We chose CSF because it interacts with the brain's interstitial fluid and factors present in CSF can influence brain microvessels indirectly, making it a more relevant proxy for MS-associated inflammation than blood (Xiang et al., 2023). Because single-cell RNA analysis (Caratis et al., 2025) revealed no or trace amounts of *CYP7B1* in the BBB cells we did not examine it further in this study. Similarly, HBMECs only expressed *HSD3B7,* which at the same time, was the strongest and most stably expressed gene in the EBI2/oxysterol pathway in all three cell types. Overall, the data showed downregulation of *HSD3B7* in HASTR, HBPC and HBMEC upon treatment with CSF from patients with MS at various time points (Fig. 2A–C). HBPCs upregulated *EBI2* and the levels of *CH25H* were largely unaffected by CSF treatment in all three cell types. Subsequently, to examine whether these effects are specific to CSF, we treated the BBB cells with a cocktail of pro-inflammatory cytokines TNFα/IL17 for the same duration (Additional Fig. 3). TNFα and IL-17 were selected not only because of their established roles in MS pathophysiology, but also because they are particularly relevant in the context of our BBB spheroids, which incorporate human astrocytes. We have previously demonstrated that TNFα and IL-17 together induce pro-inflammatory signaling in human astrocytes, and that the EBI2/oxysterol pathway modulates these responses by downregulating TNFα/IL-17-induced pro-inflammatory signaling (Elain et al., 2014; Kutryb-Zajac et al., 2022; Rutkowska et al., 2018). The data showed that the pro-inflammatory cytokines exerted faster and greater effects on gene expression in the BBB cells than the CSF. The expression of *EBI2* and *CH25H* in HASTR and HBPCs were modulated after as little as 4 h. Overall, the CSF and the pro-inflammatory cytokines downregulated *HSD3B7* while *EBI2* transcripts were upregulated in the CSF treated HBPCs and downregulated in HASTR and HBPCs.

### 2.3 VE-cadherin is modulated by the EBI2/oxysterol pathway

**Fig. 3:**
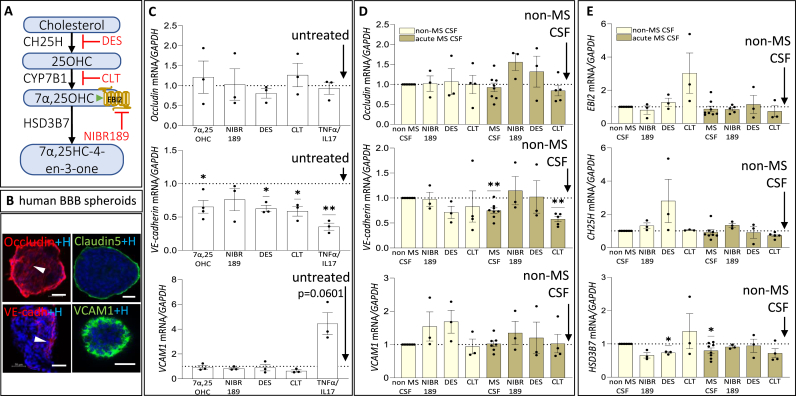
**VE-cadherin is modulated by the EBI2/oxysterol pathway.** A. 7α, 25OHC synthesis pathway with the EBI2 receptor antagonist, NIBR189, and CH25H inhibitor, desmosterol, and CYP7B1 inhibitor, clotrimazole, shown. B. Immunostaining of untreated tri-cell human BBB spheroids reveals the presence of tight junction protein occludin and claudin5, adherens junction protein VE-cadherin and adhesion protein VCAM1, indicating the establishment of tight junctions and barrier function. Scales 50 μm C. Treatment of BBB spheroids with either the EBI2 agonist, 7α,25OHC, the antagonist, NIBR189, or enzyme inhibitors, desmosterol and clotrimazole, did not affect the expression of *Occludin. The pro-inflammatory cytokines, TNFα and IL17, also did not modify its expression after 18H. The VE-cadherin levels were downregulated by all treatments except for NIBR189. The expression of VCAM1* was induced only by TNFα/IL17 treatment (p = 0.0601) and not affected by other treatments. Dotted line indicates levels in untreated spheroids to which each treatment was normalised. Data presented as mean ± SEM, one-sample *t*-test ∗p < 0.05, N = 3 independent experiments with 8–10 spheroids combined per treatment. D. CSF from patients with MS induced a downregulation of *VE-cadherin* (0.75 ± 0.05) and treatment with NIBR189 or desmosterol, rescued its downregulation. Clotrimazole, did not rescue *VE-cadherin* levels to non-MS levels (0.58 ± 0.05). Tight junction protein *occludin and adhesion marker VCAM1 were not affected by CSF. Dotted line indicates levels in non-MS CSF-treated spheroids to which all treatments were normalised. E. CSF from patients with MS did not modulate EBI2 or CH25H levels in the BBB spheroids but induced a downregulation of the degrading enzyme HSD3B7* (0.8 ± 0.08). In the presence of the CH25H inhibitor, desmosterol, spheroids treated with CSF from non-MS patients, but not CSF from patients with MS, significantly downregulated *HSD3B7* expression (0.74 ± 0.03). Dotted line indicates levels in non-MS CSF-treated spheroids to which all treatments were normalised. Data presented as mean ± SEM, one-sample *t*-test ∗p < 0.05, N = 3–8 independent experiments with 8–10 spheroids combined per treatment. Dotted line indicates levels in untreated spheroids (no CSF) to which each treatment was normalised. Data presented as mean ± SEM, one-sample *t*-test ∗∗p < 0.01, N = 3–8 independent experiments with 8–10 spheroids combined per treatment.

To investigate if the EBI2/oxysterol pathway is involved in the regulation of the BBB function in the context of MS, we built spheroids with the HASTRs, HBPCs and HBMECs, and treated them *in vitro* with CSF and various EBI2/oxysterol pathway modulating compounds (Fig. 3A and B). We investigated the effects of the above treatments on the expression of *Occludin, VE-cadherin* and *VCAM1* because these are some of the key proteins involved in maintaining the integrity and function of the BBB. VE-cadherin is an endothelial-specific adhesion molecule crucial for endothelial junction integrity and barrier stability and occludin is a critical tight junction protein essential for regulating BBB selectivity. Most importantly, it was demonstrated before that serum from patients with MS reduces the levels of VE-cadherin and Occludin *in vitro* in endothelial cells (Minagar et al., 2003). Here we extended these observations by investigating whether the CSF from patients with MS has similar effects. Treatment of the spheroids with the EBI2 agonist, 7α,25OHC, the antagonist, NIBR189, the CH25H inhibitor, desmosterol, or the CYP7B1 inhibitor, clotrimazole, did not have any effect on the expression of *Occludin* or *VCAM1* (Fig. 3C) (Clottu et al., 2017; Gessier et al., 2014; Liu et al., 2011; Lund et al., 1998; Rodríguez-Acebes et al., 2009; Rutkowska et al., 2018, Rutkowska et al., 2017, 2015; Wang and Tuohimaa, 2006). Incubation of the spheroids with a cocktail of pro-inflammatory cytokines TNFα/IL17 induced the expression of VCAM1 as was described earlier, although here the statistical significance was not reached (Lin et al., 2015). However, the expression of *VE-cadherin* was highly regulated by the EBI2/oxysterol pathway compounds (and TNFα/IL17) except for the treatment with NIBR189, the EBI2 antagonist, which did not induce downregulation of *VE-cadherin*. In mice, a single ip injection with a biostable fluoro analogue of 7α,25OHC, the EBI2 agonist, also showed a trend towards downregulation of *VE-cadherin* in the brain with no such trend observed for *Occludin* or *N-cadherin* (Additional Fig. 4**)**. When the spheroids were treated with CSF, again only *VE-cadherin* was downregulated and only in the spheroids treated with CSF from patients with MS (Fig. 3D). Co-treatment of the spheroids with the CSF from patients with MS and NIBR189, the EBI2 antagonist, or desmosterol, CH25H inhibitor, rescued the CSF induced downregulation of *VE-cadherin.* We also examined the expression of *EBI2* and *CH25H* and *CYP7B1* in the CSF treated spheroids and again found only *HSD3B7* affected by the treatments (Fig. 3E). CSF from patients with MS downregulated *HSD3B7.* A co-treatment with CSF from non-MS controls and the CH25H inhibitor, desmosterol, also downregulated *HSD3B7*.

### 2.4 EBI2 mediates the attachment of CD4^+^ T cells to the BBB spheroids *in vitro*

**Fig. 4:**
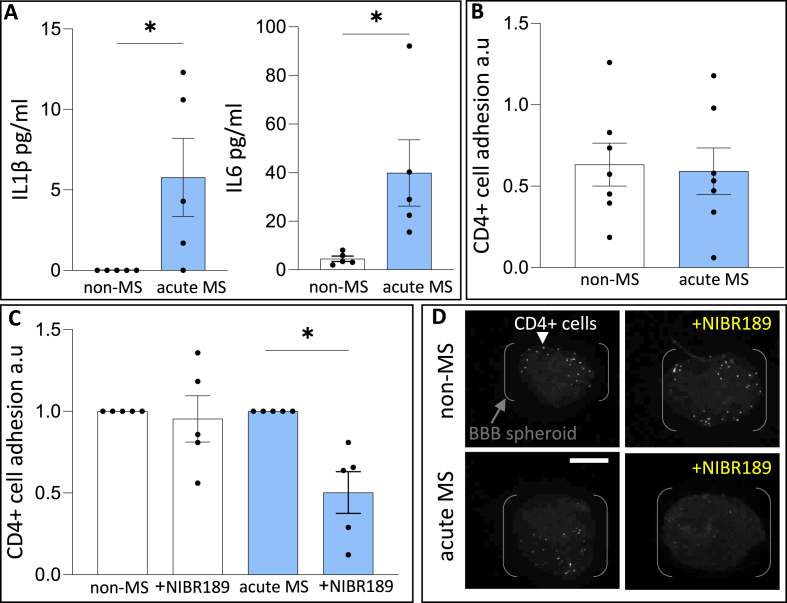
**EBI2 mediates the attachment of CD4^+^ T cells to the BBB spheroids *in vitro.*** A. The levels of secreted IL1β and IL6 cytokines were assessed in the supernatant collected from cultured CD4^+^ cells. The levels of both cytokines were significantly increase in the media collected from CD4^+^ cells from patients with MS. Unpaired *t*-test, ∗p < 0.05, N = 5 patients with MS and N = 5 non-MS controls. B. Similar number of CD4^+^ T cells from patients with MS and non-MS controls migrated and attached to the BBB spheroids. Unpaired *t*-test, p > 0.05, N = 7 patients with MS and N = 7 non-MS controls. C. Treatment of CD4^+^ T cells from non-MS patients with NIBR189 did not modulate the number of cells attaching to the spheroid. However, treatment of CD4^+^ T cells from patients with MS with NIBR189 reduced the number of cells attaching to the BBB spheroids. One-sample *t*-test, ∗p < 0.05, N = 5 independent experiments each with different patient's cells. Treatments of 4–6 spheroids per well, each spheroid was analysed separately and then pooled together within each experiment. Dots represent averaged values per well per experiment normalised to the respective control. D. Representative images of CD4^+^ T cells (white) attached to the spheroids quantified in C, scale 100 μm.

The data thus far demonstrated that CSF collected from patients with MS and the EBI2/oxysterol compounds (7α,25OHC, desmosterol, clotrimazole) regulate the expression of *VE-cadherin* and the enzyme levels in the BBB spheroids. We therefore examined the chemotaxis and attachment of CD4^+^ T cells collected from the patients with MS to the BBB spheroids alone or in the presence of the EBI2/oxysterol compounds, the EBI2 antagonist NIBR189, the CH25H inhibitor desmosterol or the CYP7B1 inhibitor, clotrimazole (Fig. 4, Additional Fig. 5). We first established that the CD4^+^ T cells isolated from patients with MS and non-MS controls retain their different functionality when cultured *in vitro*. For this purpose we measured the levels of secreted pro-inflammatory cytokines IL6 and IL1β in the cell culture supernatant. The data showed significantly increased release of IL-1β and IL-6 in CD4^+^ T cells collected from patients with MS (Fig. 4A). These findings confirmed functional differences between the CD4^+^ cell groups and indicated different molecular composition of the culture media as a result of cell-released factors. Going back to the CD4^+^ T cell adhesion assay, the data showed comparable number of CD4^+^ T cells from patients with MS and non-MS participants attached to non-treated spheroids (Fig. 4B). However, when the spheroids were treated with the EBI2 antagonist, NIBR189, significantly less CD4^+^ T cells from patients with MS attached to the BBB spheroids (Fig. 4C and D). Treatment with enzyme inhibitors, desmosterol or clotrimazole, did not have inhibitory effect on the attachment of CD4^+^ T cells to the BBB spheroids **(**Additional Fig. 5**)**.

## 3 Discussion

The data reported in this study suggests a mechanism by which increased concentrations of oxysterols in the CNS during inflammation induce downregulation of VE-cadherin, the key molecule involved in barrier formation between endothelial cells thus facilitating CNS entry of peripheral cells. The increased *CH25H* and *EBI2* transcripts inside MS plaques confirm and expend previous reports of increased EBI2 in the plaques in infiltrating lymphocytes (Wanke et al., 2017) and in glial cells (Klejbor et al., 2021). This data suggests potential implications for the pathophysiology of MS. It is possible that increased concentration of the synthesizing enzyme could elevate the levels of 7α,25OHC, potentially forming a gradient within the plaques. Such a gradient would attract EBI2-expressing peripheral cells, though further investigation would be necessary to confirm this hypothesis.

### 3.1 HSD3B7 is highly regulated in the human BBB *in vitro* spheroids model

Importantly, the data showed that CSF from patients with MS modulates the EBI2/oxysterol system in the *in vitro* human BBB model. The CSF induced a downregulation of *HSD3B7*, the enzyme responsible for degrading 7α,25OHC, which could potentially lead to an increase in 7α, 25OHC concentration. A recent study demonstrated that treatment of human BBB cells *in vitro* with TNFα indeed upregulates 25OHC (Loiola et al., 2024). Simultaneously, there was an upregulation of *EBI2*, suggesting a possible interplay between these factors. Reduced concentrations of CH25H in the CSF of patients with MS could be activating a feedback mechanism which, in response to reduced oxysterol content, may downregulate the degrading enzyme (*HSD3B7*) and at the same time upregulate *EBI2*. However, such speculations require detailed measurements of changes in the oxysterol levels and have not been investigated here. However, we indirectly confirmed our observations in the BBB spheroids co-treated with CSF and EBI2/oxysterol compounds. Desmosterol, the CH25H inhibitor, also downregulated *HSD3B7* in the spheroids. Similarly, the experiments with spheroids confirmed our observations of downregulated *HSD3B7* in the BBB cells. CSF from patients with acute MS alone downregulated *HSD3B7* in the spheroids, which could have led to an increase in 7α, 25OHC levels, potentially contributing to the maintenance of a stable oxysterol gradient. However, this possibility warrants further investigation to be definitively established. Overall, HSD3B7 seems highly regulated in the BBB cells by the CSF of patients with MS.

### 3.2 Study limitation – *in vitro* incubation of BBB cells and spheroids with CSF

An important caveat to this part of our study is the use of CSF directly on the BBB spheroids. Under normal conditions, CSF does not contact the BBB. However, our goal was to investigate the influence of CNS-derived soluble factors present during MS relapse. Since these factors are cleared into and accumulate in the CSF, this fluid serves as a rich source for capturing the inflammatory milieu of the CNS. Compared to peripheral blood, CSF provides a more concentrated and CNS-specific snapshot of disease-associated mediators. By applying CSF directly to our *in vitro* BBB model, we sought to examine the effects of this complex mixture on barrier properties in a simplified and controlled setting. While this approach does not replicate the anatomical directionality of CNS-to-BBB signalling, it offers a valuable proxy to explore the biological impact of CNS-derived factors on BBB integrity. Importantly, CSF is the closest accessible fluid to CNS tissue that can be obtained from living patients, making it a practical and informative tool for translational studies. Although this setup has limitations, it provides meaningful insights into how inflammatory mediators present in the CNS during MS may influence BBB structure and function.

### 3.3 EBI2/oxysterol signalling modulates VE-cadherin in MS

VE-cadherin is a key component of adherens junctions involved in endothelial cell-cell adhesion, thereby contributing to the formation of a tight and selective barrier (Wary and Sreedhar, 2017). Here, we found that expression of *VE-cadherin* is not only downregulated by CSF from patients with MS, but that it is also modulated by the EBI2/oxysterol system, possibly opening up new ways to modulate the brain vasculature also in MS. Our study confirms and builds on the findings of Minagar and colleagues (Minagar et al., 2003) who demonstrated that serum from patients with MS reduces VE-cadherin and occludin levels in endothelial cells *in vitro*. Here, we demonstrated that CSF from patients with MS has similar effects on the expression of VE-cadherin. Treatment of the BBB spheroids with the CYP7B1 inhibitor, clotrimazole, yielded similar effects to treatment with CSF from patients with MS, that is downregulation of *VE-cadherin*, although this is most likely due to only trace expression of CYP7B1 in the human BBB cells (Caratis et al., 2025). Moreover, clotrimazole is not CYP7B1 specific and acts on many pathways including other cytochrome P450 enzymes (Shord et al., 2010). However, co-treatment of the spheroids with CSF from patients with MS and with either the EBI2 antagonist, NIBR189, or the CH25H inhibitor, desmosterol, rescued the CSF induced downregulation of *VE-cadherin* suggesting involvement of the EBI2/oxysterol system in VE-cadherin regulation during MS episode. The downregulation of *VE-cadherin* observed after treatment with a cocktail of pro-inflammatory cytokines TNFα/IL17 may suggest that inflammatory stimuli could generally reduce VE-cadherin expression, potentially contributing to the disruption of the barrier during inflammation. This data is in line with a recent report of increased BBB permeability and reduction of claudin-5 in TNFα treaded human *in vitro* BBB model (Loiola et al., 2024).

### 3.4 Study limitations – limited complexity of the human BBB *in vitro* model and small sample size in *in vitro* experiments

Our findings should be interpreted with caution due to certain limitations. The sample size in the experiments with spheroids was relatively low, which may impact the robustness of the conclusions. Moreover, while our *in vitro* model provides valuable insights, it does not fully replicate the complexity of the BBB *in vivo*. Future studies with larger sample sizes and complementary *in vivo* approaches will be essential to validate these observations.

### 3.5 EBI2/oxysterol-mediated, VE-cadherin-specific downregulation in a human *in vitro* BBB model and in mice

A recent study in CH25H knock-in mice demonstrated the key role of CH25H in brain endothelial cells in the EAE model (Ruiz et al., 2023). Earlier, Wanke and colleagues (Wanke et al., 2017) found upregulated CNS expression of *ch25h* in EAE and Chalmin and colleagues (Chalmin et al., 2015) demonstrated that CH25H modulates the inflammatory response in EAE by controlling lipid metabolism and the production of oxysterols. Another recent study demonstrated upregulated expression of *Ch25h* and levels of 25OHC in the brains of LPS-treated mice (Romero et al., 2024). Here, the involvement of the EBI2/oxysterol system in the regulation of *VE-cadherin* was demonstrated by treating the BBB spheroids with EBI2 compounds. 7α, 25OHC and both enzyme inhibitors, desmosterol and clotrimazole, downregulated *VE-cadherin*. Only the EBI2 antagonist, NIBR189, did not downregulate its levels indicating an EBI2-mediated mechanism. Our study in mice provided partial support for our observations in human cells, indicating a possible trend toward *VE-cadherin* downregulation in the mouse brain following ip injection with a biostable synthetic analogue of 7α,25OHC. Such trend was not found for occludin or N-cadherin indicating VE-cadherin specific effects of 7α,25OHC.

### 3.6 Study limitation – species differences in EBI2/oxysterol-related gene expression in the mouse and human BBB cells

The results in mice need to be interpreted with caution as we have observed significant species differences in EBI2/oxysterol-related gene expression in the mouse and human BBB cells (Caratis et al., 2025). It is also important to emphasize the differences in enzyme expression across human tissues and cell types. Our findings show high *CYP7B1* expression in human brain lysates and isolated brain microvessels, while no or trace levels of this enzyme were detected in the BBB cells (HBMECs, HASTRs and HBPCs). These differences are likely due to the involvement of distinct cell types. For instance, microglia in the brain and brain microvessels are most likely responsible for CYP7B1 production. Our BBB *in vitro* model does not include microglia, which explains only trace expression of the enzyme. Indeed, it has been shown previously that not all cell types express all three enzymes. For example, in lymphoid tissues, the stromal cells increase 7α, 25OHC levels by producing CH25H and CYP7B1, while follicular dendritic cells decrease its levels by producing HSD3B7 (Yi et al., 2012).

### 3.7 EBI2 receptor signalling mediates T cell attachment to the human BBB *in vitro*

The data prompted us to consider whether oxysterols, known to increase during inflammation (Crick et al., 2017), might induce downregulation of *VE-cadherin* in endothelial cells, potentially facilitating the migration of EBI2-expressing immune cells to sites of injury within the CNS. To partially answer this question, we examined the chemotaxis and attachment to the BBB of CD4^+^ T cells collected from patients with MS and non-MS controls. While the T cells from patients with MS and non-MS controls migrated and attached to the untreated BBB spheroids to a similar extent, inhibition of EBI2 signalling with the antagonist NIBR189, reduced the attachment to the spheroid of the T cells collected from patients with acute MS. Because our data showed that NIBR189 does not exert effects on the expression of EBI2/oxysterol related genes in the BBB spheroids, irrespective of the presence of the CSF, we reasoned that the observed inhibitory effects are mediated via EBI2 expressed in the T cells and not the BBB spheroids. Indeed, a previous study demonstrated that NIBR189 blocks migration of CD4^+^ T cells collected from patients with MS in a transwell assay (Clottu et al., 2017) but that study did not investigate migration towards and attachment to the BBB, which are of key importance in the pathophysiology of MS.

The EBI2 antagonist, NIBR189, uniquely maintained *VE-cadherin* levels in both untreated spheroids and those treated with CSF from patients with MS. Furthermore, only NIBR189 effectively inhibited the attachment of CD4^+^ T cells to the BBB. These findings highlight an EBI2-dependent mechanism. In conclusion, the data suggests that potential therapeutic strategies might benefit from focusing on modulating the EBI2 receptor rather than enzyme levels. This approach could, in theory, help prevent CNS infiltration by autoreactive immune cells that express high levels of EBI2, though further research is needed to explore this possibility.

## 4 Materials and methods

### 4.1 Human brains, CSF and blood collection

All human participants gave their written informed consent in accordance with the Declaration of Helsinki. The Independent Bioethics Committee For Scientific Research at Medical University of Gdańsk (Poland) approved the study under the licence number: NKBBN/457/2019. Frozen human brains from patients with and without MS were sourced from the Rocky Mountain MS Centre Tissue Bank (Englewood, CO, United States) after obtaining approval from the Medical University of Gdańsk (Poland) bioethics committee number NKBBN/253/2018. Blood and CSF samples (lumbar puncture) were collected from patients admitted to the Adult Neurology Clinic at the University Clinical Centre in Gdańsk, Poland. Sample collection, processing and storage was done according to procedures reported earlier (Miller et al., 2016, 2017). All subjects underwent both procedures, prospectively, during the routine diagnostic process (Table 1).

**Table 1:** Information on patients’ samples.

Group	Nr. of patients	Mean age ± SD (years)	Male (M), Female (F)
**MS**	N = 17	29.11 ± 6.02	M = 7; F = 11
**Non-MS**	N = 14	38.14 ± 2.98	M = 3; F = 11

Based on the initial clinical diagnosis, follow-up period and the presence of CSF oligoclonal bands, samples were enrolled into the MS group, which comprised patients diagnosed with MS from whom material was collected during first acute neurological episode (N = 16); non-treated patient with MS during relapse (N = 1). Diagnosis of MS was made in compliance with 2017 McDonald criteria **(**Additional Table 2**)** (Thompson et al., 2018). In all cases of the acute neurological episode, the samples were collected during the first clinical episode and before administering glucocorticosteroids (“acute MS” samples) or, in one case, during 24 h after 1 g of methylprednisolone. The control group (“non-MS”) included volunteers (only blood collection) and patients with non-inflammatory diseases of the CNS, mainly with headache, migraine, one with idiopathic intracranial hypertension, one with normal pressure hydrocephalus, and one with spondylotic myelopathy. After collection, the CSF was centrifuged, aliquoted, and frozen at −80 °C, whole blood was processed for CD4^+^ cell isolation (see below), centrifuged, aliquoted, and frozen at −80 °C. All *in vitro* experiments were performed with pooled samples of CSF according to the patients’ diagnosis. The CSF and plasma samples were used earlier by us in a study investigating adenosine deaminase activity *in vitro* (Kutryb-Zajac et al., 2022) and the expression and signalling of proton-sensing receptors TDAG8, GPR4 and OGR1 in organotypic cerebellar slices (Caratis et al., 2024).

### 4.2 CD4^+^ T cell isolation

Whole blood was collected from patients in order to isolate CD4^+^ T cells using human StraightFrom® Whole Blood CD4 MicroBeads (130-090-877, Miltenyi Biotec). Briefly, after whole blood collection, 50 μl of microbeads were used per 1 ml of whole blood and incubated at 4 C for 15 min. The magnetically labelled cells were then applied to a column (130-093-545, Miltenyi Biotec) and placed in a magnetic field (130-042-301). 4 ml of the elution buffer (130-093-545, Miltenyi Biotec) was used to flush out the labelled cells from the column, outside the magnetic field. Isolated CD4^+^ T cells were then centrifuged at 1300 rpm for 5 min at room temperature (RT) and resuspended at 10ˆ7 cells/ml in cold “freezing” media (90 % FBS+10 %DMSO). Cells were stored in liquid nitrogen until use. For migration experiments, cells were thawed the day before the experiment and resuspended at 10ˆ6 cells/ml in RPMI with 1 mM glutamax and 1 % penicillin/streptomycin (pen/strep). Supernatant from untreated CD4^+^ cells was collected for quantification of cytokine levels with ELISA (see details below).

### 4.3 Human brain samples and microvessel isolation

Frozen human brains were used for RT-qPCR and WB analyses of brain homogenates as well as immunostaining, WB and RT-qPCR analysis on isolated brain microvessels. Brains of patients with MS (N = 6) were all female with a history of the disease (age: 64.33±19.40 years). Brains from non-MS individuals were mainly male (N = 5; 5 male, 1 female; age: 62±24.90 years). Brain regions used were based on the autopsy report and the location of visible plaques (periventricular region). Brain regions used from control were selected to match the ones used from MS brain **(**Additional Table 3). Microvessels were isolated from frozen brains based on an existing protocol (Hartz et al., 2018). Briefly, meninges were removed and the WM was cut out. Then, the tissue was cut on ice with a scalpel and transferred to a dounce homogeniser. Brain slices were homogenised in cold DPBS with calcium and magnesium (21-030-CV, Corning), pH 7.4, 4 °C. The homogenates were moved to 50 ml tubes and 30 % polysucrose 400 (P7798-100g, Merck) in DPBS was added in equal volume for a final concentration of 16.5 % polysucrose 400. After vigorous shaking, the homogenates were centrifuged at 5800 g for 15 min at 4 °C (fixed angle rotor, medium deceleration speed). The pellets were resuspended in 2 ml of DPBS and were then filtered through a 300 μm mesh. The mesh was washed with 50 ml of DPBS. The flow-through was filtered through a 30 μm cell strainer twice and the mesh was washed twice with DPBS before discarding the filtrate. The filter was turned upside-down and washed twice with DPBS to collect the capillaries. After collection, the capillaries were centrifuged twice in DPBS at 1500 rpm for 3 min at 4 °C. The microvessels were then resuspended in DPBS for subsequent analysis.

### 4.4 Pharmacokinetic *in vivo* mouse experiment

The protocol was approved by the Local Ethical Committee for Animal Experiments in Bydgoszcz (Poland) following national and international guidelines and ethical regulations under permission number Nr 34/2023. All mice were maintained in a pathogen-free animal facility under standard animal room conditions: temperature 20–23°C; humidity 55 %–60 %; 12-h light/dark cycle and air exchange 15 times per hour. A different set of data obtained from this study was published earlier (Konieczna-Wolska et al., 2024). Forty 6–9 week old male C57BL/6 mice were used in the experiment with 8 mice per treatment group. Mice were injected subcutaneously (sc) with either vehicle (canola oil) or 100 mg/kg CF3-7α, 25OHC and sacrificed after 6, 12, 24 or 48 h after injection. The brains were snap-frozen separately and kept for subsequent qPCR analysis.

### 4.5 Cell culture

The human astrocytes clone 35 (HASTR) were immortalized by transduction with the SV40 large T antigen, which promotes continued cell proliferation by inactivating tumor suppressor proteins p53 and retinoblastoma protein (Rb) (Furihata et al., 2016). The human brain vascular pericytes clone 37 (HBPC) were immortalized via transduction with the human telomerase reverse transcriptase (hTERT) gene, which extends cell lifespan by maintaining telomere length and preventing senescence (Umehara et al., 2018). The human brain microvascular endothelial cells clone 18 (HBMEC) were immortalized through transfection with the SV40 T antigen, similar to the HASTR cells, which prevents senescence by interfering with the p53 and Rb pathways (Kamiichi et al., 2012). The HASTR media consisted of DMEM supplemented with 10 % FBS, 1 % N2 supplement-A (07152, Stemcell) and 1 % pen/strep (Furihata et al., 2016). HBPC were cultured in pericytes media (1201, ScienCell)(Umehara et al., 2018). HBMEC's growth media was EBM-2 BM (Lonza, CC-3162), without gentamicin, with 10 mM GlutaMax and 1 % pen/strep (Kamiichi et al., 2012). All cells were grown and cultured at 33 °C, 5 % CO2. 4 mg/ml of blasticidin S was added to each culture to keep them immortalized until experiments. Cells where then plated in 6-well plates, 150 000 cells/well, two wells per condition and moved to 37 °C, 5 % CO_2_, without blasticidin S, to allow them to differentiate. They were then each separately treated with serum-free media or 10 ng/ml TNFα (Bio-techne, 210-TA) + 50 ng/ml IL17 (Bio-techne, 317-ILB) for 4, 18 or 24 h (h) as was described before (Elain et al., 2014; Kutryb-Zajac et al., 2022). Separately, cells were also treated with 10 % pooled CSF from patients with MS or non-MS for 4, 18 or 24 h. The cells were then washed in PBS and collected in 400 μl fenozol for PCR. TNFα/IL17 condition was compared to untreated cells and MS to non-MS.

### 4.6 Spheroid treatment

The spheroid model was built according to our validated protocol (Isogai et al., 2022; Kitamura et al., 2021). Additional validation and characterisation of the spheroids used in the current study is shown in Additional Fig. 6, which shows that the spheroid structure resembles brain microvessels with the endothelial cells forming a tight outer layer with interspersed pericyte and astrocyte endfeet (Additional Fig. 6A and B**)**. The spheroids are a suitable model to investigate the effects of pro-inflammatory signalling on BBB permeability and protein (occludin and VE-cadherin) expression (Additional Fig. 6C**)**. The growth media for spheroid is the EBM-2 BM, without gentamicin, with 10 mM GlutaMax, 1 % pen/strep and supplemented with 0,48 mg/ml methylcellulose 400 (M0262-100g, Merck). Briefly, on day one, HASTRs were plated in 96-well V-bottom plates (249935, ThermoFisher) at a concentration of 3,5.10ˆ4 cells/ml (50 μl). The following day, 1,0.10ˆ4 cells/ml (50 μl) of HBPC were added to the HASTR. Finally, HBMEC were plated, 1,5.10ˆ4 cells/ml (50 μl) on day three. Spheroids were used for experiments three to five days after adding HBMEC. All spheroids, from day one, were grown at 37 °C, 5 % CO_2_ without blasticidin S. During experiments, the media was replaced with serum-free spheroid media or, for migration, RPMI with 1 mM glutamax and 1 % pen/strep. Spheroids were treated with different compounds for 18 h: TNFα+IL17, 1 μM 7α,25OHC, 10 μM NIBR189, 1 μM clotrimazole (C6019, Sigma), 2.5 μl/ml desmosterol (700060P, Sigma), 100 ng/ml LPS (L4391, Sigma) and/or 10 % CSF from patients (see Additional Table 2 for details). After 18h treatment (8–10 spheroids per condition per experiment), the spheroids were collected in 400 μl fenozol for PCR analysis or in PBS for staining (two spheroids per antibody).

### 4.7 Dextran permeability assay

After 18h treatment with LPS or serum-free media, spheroids were collected and washed with PBS. Spheroids were then incubated in fluorescein isothiocyanate-dextran 70 kDa (46945, Sigma), 75 μg/ml for 12h at 37 °C. After incubation, spheroids were washed twice in PBS, fix in 4 % PFA for 15 min, washed again with PBS and then mounted. Z-stacks were taken with a confocal Zeiss LSM880 microscope with a 2.5 μm step. Analysis was performed using the ImageJ software, measuring the fluorescence intensity from maximum intensity projection images (background intensity was subtracted).

### 4.8 Adhesion assay

CD4^+^ cells isolated from patients were used for migration experiments. Cells were starved beforehand in RPMI media with 2 mM glutamax and 1 % pen/strep and stained with CellTracker Deep Red (C34565, Thermofisher) according to the manufacturer's instructions. For the EBI2 antagonist NIBR189 (1 mM), cells were pre-incubated for 30 min before being added to the spheroids. Roughly 2000 CD4^+^ T cells were added to every spheroid for 48 h, 4 spheroids/patient/treatment. The media during migration contained different treatments including 1 mM NIBR189, 1 μM clotrimazole (C6019, Sigma) or 2.5 μl/ml desmosterol (700060P, Sigma). After migration, spheroids were collected, washed in DPBS (+), fixed 10 min in 4 % PFA and mounted. Z-stacks were taken with a confocal Zeiss LSM880 microscope with 2.5 μm step. Analysis was performed using the ImageJ software, measuring the fluorescence intensity from maximum intensity projection images (background intensity was subtracted). Treatment conditions were normalised to their respective untreated condition, i.e. treated with cells from the same patient, for each experiment.

### 4.9 Enzyme-linked immunosorbent assay (ELISA)

ELISAs with CSF and serum were performed using human cholesterol-25-hydroxylase (#MBS2706087, MyBioSource). Serum samples were diluted 1:2 in PBS before the assay. Briefly, 100 μl of standard or undiluted CSF or serum sample was added to the plate and then incubated for 1 h at 37 °C. 100 μl of reagent A was then added to each well and incubated 1 h at 37 °C. The wells were then emptied and washed three times with wash buffer. 100 μl of reagent B was then added and plates incubated 30 min at 37 °C. The wells were washed once again. Subsequently, 90 μl of substrate solution was added and incubated for 15 min (until blue colour developed). To stop the reaction, 50 μl of stop solution was added. ELISAs were also performed on the supernatant collected from CD4^+^ T cells using DuoSet ELISA human IL-1b (DY201) and human IL-6 (DY206). Briefly, plates were coated overnight with the capture antibody at RT, then washed three times with wash buffer (0.05 % Tween-20 in PBS). Plates were then blocked with reagent diluent (1 % BSA in PBS) for 1 h RT and washed three times. 100 μl of supernatant from the isolated CD4^+^ T cells was added to the plates for 2 h, then washed three times. Afterwards, detection antibody was added for 2 h and plates were washed three times. Finally, Streptavidin-HRP was added to the plates for 20 min and washed three times, then 100 μl of TMB substrate solution (34021, ThermoFisher) was added for 20 min followed by 100 μl of 1 mol/L H2SO4 to stop the reaction. Optical density for all ELISA assays was read at 450 nm on VICTOR Nivo plate reader (PerkinElmer).

### 4.10 Real-time quantitative polymerase chain reaction (RT-qPCR)

Fragments of frozen human brains were crushed in liquid nitrogen to obtain a powder which was then resuspended in fenozol for RNA isolation of brain homogenates. Previously isolated microvessels were also resuspended in fenozol. Total RNA was isolated with fenozol with total RNA mini plus kit (036–100, AA Biotech). Briefly, fenozol samples were heated for 5 min at 50 °C, then 150 μl of RNAse free water was added, vortexed and incubated for 5 min. Samples were then centrifuged for 15 min at 12 000 rpm. 400 μl of supernatant was collected, mixed with 400 μl of isopropanol and applied to the column. Columns were then centrifuged for 1 min at 12 000 rpm. Eluted volume was discarded and columns were washed three times in wash buffer. Finally, 50 μl of RNAse free water was added to the column, incubated for 2 min and then centrifuged to elute the obtained cDNA. Purity and quantity were assessed using the PerkinElmer VICTOR Nivo plate reader. The cDNA synthesis was performed with the high-capacity cDNA reverse transcription kit (4368814, ThermoFisher) using the following programme: 10 min at 25 °C followed by 120 min at 37 °C and 5 s at 85 °C. RT-qPCR was performed with the TaqMan fast advanced master mix (4444557, ThermoFisher) on the LightCycler 480 (Roche) according to the manufacturer's protocol. The following FAM dye-labelled Taqman (Applied Biosystems) human primers were used: β-actin (Hs03023943_g1), GAPDH (Hs02786624_g1), EBI2 (Hs00270639_s1), CH25H (Hs02379634_s1), CYP7B1 (Hs_01046431_m1), HSD3B7 (Hs00986913_g1), Occludin (Hs05465837_g1), N-cadherin (Hs00983056_m1), VE-cadherin (Hs00901465_m1) and VCAM1 (Hs01003372_m1) and mouse primers: GAPDH (Mm99999915_g1), Occludin (Mm00500912_m1), N-cadherin (Mm01162490_m1) and VE-cadherin (Mm00486938_m1), VCAM1 (Hs01003372_m1) and claudin5 (Hs00533949_s1). The relative gene expression was determined using the ΔCt and ΔΔCt method based on absolute quantification after normalisation to the housekeeping gene.

### 4.11 Western blot

Frozen human brains were homogenised with a Dounce homogeniser in RIPA buffer with protease inhibitor (700 μl/100 mg of tissue). Isolated microvessels were resuspended in RIPA buffer with protease inhibitor. Samples were sonicated with a bandelin sonicator (1.5 M) four times for 10 s at an amplitude of 75 % and one final time for 10 s at 85 % amplitude. The samples were equalised and mixed with 1x Laemmli sample buffer (1610747, Bio-Rad) + 10 % β-mercaptoethanol, then incubated for 10 min at 70 °C. The protein samples were separated on 8 %, 10 % or 12.5 % SDS-polyacrylamide gels for 2 h, 125 mA and transferred to a PVDF membrane (IPFL00005, Merck) for 1 h, 20 V. The membrane was blocked for 1 h at RT in PBS with 0.1 % Tween-20 (PBS-T), 5 % non-fat milk and then incubated with primary antibody overnight at 4 °C. The membrane was washed 3 times for 10 min and then incubated with secondary antibodies for 1 h at RT in the dark. After three 10-min washes, the membrane was rinsed with water and read with WB scanner Odyssey. Band intensity was quantified using the software ImageJ and the protein of interest was normalised to actin or α-tubulin. Primary antibodies used were: rabbit Occludin 1:500 (RRID: AB_2533468, 40–4700, ThermoFisher), mouse N-cadherin 1:500 (RRID: AB_2313779, 333900, ThermoFisher), rabbit VE-cadherin 1:200 (RRID: AB_2533243, 36–1900, ThermoFisher), rabbit Claudin-5 1:1000 (ZRB2487, Sigma) rabbit α-tubulin 1:5000 (RRID: AB_2546920, PA5-29444, ThermoFisher) and mouse actin 1:5000 (RRID: AB_476744, A5441, Sigma) in PBS-T. Secondary antibodies used were donkey anti-mouse Alexa 790 (RRID: AB_2340870, 715-655-150, JacksonImmuno), goat anti-rabbit Alexa 680 (RRID: AB_2338085, 111-625-144, JacksonImmuno), 1:15000 in PBS-T.

### 4.12 Immunostaining

The human cells were separately plated on collagen I coated 8-well ibidi plates, 50 000 cells per well, and incubated at 37°C for two to three days before subsequent staining. After isolation, capillaries were plated in PBS on poly-D-lysine coated 8-well ibidi plates and left to adhere for 2 h in the incubator at 37 °C and 5 % CO_2_. The media was removed and the capillaries were left to air dry for 30 min. The capillaries and the cells were washed once in PBS, then fixed in 4 % PFA for 10 min. The PFA was removed and 50 μl of ice-cold methanol was added for 1 min. Capillaries and cells were washed twice with PBS at RT and then were blocked for 1 h in 0.5 % NGS, 1 % BSA and 0.1 % Tween20 in PBS. They were then incubated overnight at 4 °C in 0.5 % BSA and 0.05 % Tween20 in PBS with the primary antibodies. The capillaries and the cells were washed twice with PBS, then once in 0.5 % BSA +0.05 % Tween20 in PBS for 10 min. As for the spheroid, they were collected for staining and washed in PBS before 15 min fixation in 4 % PFA. Spheroids were washed twice in PBS before blocking in 10 % BSA, 0.5 % Triton-X and 1 % NGS for 1 h. Antibody solution for spheroids consisted of 2 % BSA and 0.1 % Triton-X. Capillaries, cells and spheroids were incubated with secondary antibodies and Hoechst for 1 h at RT in the dark in their respective antibody solution. They were then washed three times in PBS and once in ddH2O for 10 min then air-dried, mounted with Keiser's gelatine and imaged with a confocal microscope (Zeiss). Validated and published antibodies were used for staining. The following antibodies were used at 1:100 unless stated otherwise: mouse EBI2 (1:50, 57C9B51C9) (Chalmin et al., 2015; Clottu et al., 2017; Klejbor et al., 2021; Preuss et al., 2014; Rutkowska et al., 2015) provided by Novartis, rabbit polyclonal CH25H (600-401-MM8, ThermoFisher)(Caratis et al., 2025), mouse monoclonal CYP7B1 (OTI1G7) (TA807549, ThermoFisher)(Caratis et al., 2025), rabbit polyclonal HSD3B7 (RRID: AB_10856786, BS-2366R, ThermoFisher) (Caratis et al., 2025), mouse N-cadherin (RRID: AB_2313779, 333900, ThermoFisher)(Caratis et al., 2025; Wang et al., 2020), rabbit VE-cadherin (RRID: AB_2533243, 36–1900, Thermofisher)(Alshaikh et al., 2023; Sheppard et al., 2023; van der Stoel et al., 2023), rabbit Occludin (RRID: AB_2533468, 40–4700, ThermoFisher) (Salman et al., 2020)), rabbit VCAM1 (AB_2809259, MA5-31965, ThermoFisher) (Wu et al., 2024), goat polyclonal PDGFRβ (RRID: AB_355339, AF385, R&D Systems) (Gao et al., 2024), rabbit monoclonal GFAP (RRID: AB_2631098, 12389, Cell Signalling) (Smiley et al., 2025), mouse monoclonal CD31 (RRID: AB_10596359, BMS137, ThermoFisher) (Jalili-Firoozinezhad et al., 2018) and rabbit Claudin-5 (ZRB2487, Sigma). The following secondary antibodies were used (1:500): sheep anti-mouse IgG (H + L) Alexa Fluor 647 (RRID: AB_2340340, 515-605-003, Jackson Immunoresearch), donkey anti-rabbit IgG (H + L) Alexa Fluor 488 (AB_2313584, 711-545-152, Jackson Immunoresearch) and Hoechst 33342 (H1399, ThermoFisher).

### 4.13 Mass spectrometry

#### 4.13.1 Sample preparation for LC-MS/MS

All clinical and pooled samples for library generation and quantitative analysis were prepared according to the standard FASP protocol on the 10 kDa membrane and tryptic digestion (Wiśniewski et al., 2009). To construct an inclusive spectral library, a lysed pooled sample of A) all clinical samples used for analysis in this work and B) CSF samples from our collection were processed with alternative methods:

1) In-gel separation using an automated SageELF system (Sage Science) as previously described (Musiał et al., 2023). The procedure was carried out on 3 % agarose gel cassettes. The yielded 13 fractions were digested using the FASP protocol;
2) Immunodepletion with Multiple Affinity Removal Spin Cartridge Human 14 (MARS-14) kit (Agilent Technologies, Santa Clara, CA) according to manufacturer's protocol. The procedure was repeated 10 times, and the resulting fractions were combined and subjected to ultrafiltration by centrifugation on the 10 kDa Amicon membrane to exchange the buffer (from the buffer used in the MARS kit when eluting with 50 mM Tris-HCl pH 8) and then subjected to the FASP procedure;
3) High pH separation: the digested pooled samples were fractionated with a High pH Reversed-Phase Peptide Fractionation Kit (Thermo Fisher Scientific), which was performed following the manufacturer's instructions (Cat No. 84868, Thermo Fisher Scientific).

Before LC-MS/MS analysis, all samples were subjected to final purification using the StageTips technique on the C18 phase (Musiał et al., 2023).

### 4.14 Mass spectrometry analysis

Spectrum registration was performed on a TripleTOF 5600+ (Sciex Framingham, MA, USA) mass spectrometer coupled with Ekspert MicroLC 200 Plus System (Eksigent, Redwood City, CA, USA) chromatography system. All chromatographic separations were performed on the ChromXP C18CL column (3 μm, 120 Å, 150 × 0.3 mm). The chromatographic gradient for each MS run was 20–60 % B (solvent A: 0 % aqueous solution, 0.1 % formic acid; solvent B: 100 % acetonitrile, 0.1 % formic acid) for 60 min. The whole system was controlled by SCIEX Analyst TF 1.7.1 software. Measurements for the spectral library were acquired in the data-dependent acquisition (DDA) mode. Each cycle of the applied DDA method comprised an accumulation of precursor spectra in 100 ms in the range of 400–1200 m/z followed by accumulation of top 20 precursor's product ion spectra in 50 ms in the range of 100–1800 m/z, resulting in a total cycle time of 1.15 s. Formerly fragmented precursor ions were dynamically excluded.

### 4.15 Quantitative analysis

SWATH-MS and variable window acquisition were used for quantitative analysis. The precursor mass range was from 400 to 1200 m/z, and 25 variable windows were created using SwathTUNER software (Zhang et al., 2015). The scan for product ions was between 100 and 1800 m/z in 40.014 ms; the total cycle time was 10999 s. The spectra were acquired in three technical repetitions in the data-independent acquisition mode DIA (Lewandowska et al., 2021). Quantitative analysis was performed in PeakView 2.2 software (SCIEX), and the spectra library was constructed using ProteinPilot 4.5 software (Sciex, Homo sapiens database, UniProt data). All clinical sample spectra recorded in the DIA format were processed against the created library and SWATH data were generated.

The mass spectrometry proteomics data have been deposited to the ProteomeXchange Consortium (http://proteomecentral.proteomexchange.org) via the PRIDE partner repository (Perez-Riverol et al., 2022) with the dataset identifier PXD055641.

### 4.16 Statistical and enrichment data analysis

To determine proteins with statistically significant changes in levels, the data were processed in the MarkerView 1.2.1.1 software (Sciex), normalised with the total area sums (TAS) approach, and exported to Perseus 1.6.13 software (MaxQuant), where statistical tests were performed (log 2(x), q-value, and Fold Change FC). Based on the data obtained, a bioinformatics analysis was performed using the STRING 11.5 serwer (Szklarczyk et al., 2019). Data visualisation was performed in Cytoscape 3.9.1 and SRPlot (Tang et al., 2023).

### 4.17 Statistical analysis

Statistical analysis was performed using GraphPad Prism 10. Normality was assumed with the Shapiro-Wilk test when p > 0.05. Under the normality assumption, student t-tests for comparison of two groups were performed or one-sample t-tests when data was normalised in-between experiments. When p < 0.05 with the Shapiro-Wilk test, non-parametric tests were used. Wilcoxon non-parametric *t*-test was performed to compare data to a normalised control group in-between experiments. For comparison of more than two groups with no normalisation in between experiments, one-way ANOVA with Sidaks post-hoc test was performed, non-parametric Kruskal-Wallis test with Dunn's multiple comparisons post hoc tests was performed. Data are presented as mean ± standard error of the mean (SEM). When p-value <0.1, values are shown in the figures. Significant comparisons are presented with asterisks: ∗p < 0.05, ∗∗p < 0.01, ∗∗ ∗p < 0.001, ∗∗∗∗p < 0.0001. Grubbs' test was performed to identify and remove outliers.

## CRediT authorship contribution statement

**Fionä Caratis:** Writing – review & editing, Visualization, Methodology, Investigation, Formal analysis, Conceptualization. **Inez Mruk:** Visualization, Methodology, Investigation, Formal analysis. **Klaudia Konieczna-Wolska:** Writing – review & editing, Methodology, Investigation. **Bartłomiej Rojek:** Writing – review & editing, Methodology, Investigation. **Marek Hałas:** Writing – review & editing, Methodology, Investigation. **Paulina Czaplewska:** Validation, Supervision, Resources, Methodology, Formal analysis, Data curation. **Bartosz Karaszewski:** Writing – review & editing, Supervision, Resources, Methodology. **Tomomi Furihata:** Writing – review & editing, Resources, Methodology. **Aleksandra Rutkowska:** Writing – review & editing, Writing – original draft, Visualization, Supervision, Resources, Project administration, Methodology, Investigation, Funding acquisition, Formal analysis, Conceptualization.

## Declarations

### Ethics approval and consent to participate

The Independent Bioethics Committee For Scientific Research at Medical University of Gdańsk (Poland) approved the human participant study under the licence number: NKBBN/457/2019.

Non-MS frozen human brains were provided by the Rocky Mountain MS Centre Tissue Bank (Englewood, CO, United States) with approval from the Medical University of Gdańsk (Poland) bioethics committee (NKBBN/253/2018).

The mouse study was approved by the Local Ethical Committee for Animal Experiments in Bydgoszcz (Poland) following national and international guidelines and ethical regulations under permission number Nr 34/2023.

## Availability of data and materials

The mass spectrometry proteomics data supporting the conclusions of this article have been deposited to the ProteomeXchange Consortium via the PRIDE partner repository with the dataset identifier PXD055641 http://www.ebi.ac.uk/pride. All other data analysed during the current study are available from the corresponding author on reasonable request.

## Funding

This research was funded by the 10.13039/501100004442National Science Centre, Poland. Grant registration nr. 2019/33/B/NZ4/03000.

## Declaration of competing interest

The authors declare the following financial interests/personal relationships which may be considered as potential competing interests:

Aleksandra Rutkowska reports financial support was provided by National Science Centre Poland. Aleksandra Rutkowska reports a relationship with National Science Centre Poland that includes: funding grants. Aleksandra Rutkowska and Bartosz Karaszewski have patent #EP24191137.9 pending. If there are other authors, they declare that they have no known competing financial interests or personal relationships that could have appeared to influence the work reported in this paper.

## Data Availability

Data will be made available on request.
