# Supplementary material for: EBI2-oxysterol signalling regulates VE-cadherin expression and multiple sclerosis CD4+ T cell attachment to a human tri-cell spheroid blood-brain barrier model
Source: Brain Behav Immun Health. 2025 Jun 20;47:101045. doi: 10.1016/j.bbih.2025.101045 (PMC12246718; doi:10.1016/j.bbih.2025.101045)
Supplement: Multimedia component 1 [file mmc1.docx]

| **UniProt Name** | **UniProt ID** | **Log Fold Change** | **Fold Change** | **Student's T-test p-value** | **Student's T-test q-value** |  |  |  |
| --- | --- | --- | --- | --- | --- | --- | --- | --- |
| Alpha-2-HS-glycoprotein | P02765 | -2.7286 | 0.1509 | 6.0E-08 | 0.0000 |  |  |  |
| Antithrombin-III | P01008 | -1.9665 | 0.2559 | 1.6E-07 | 0.0000 |  |  |  |
| Chromogranin-A | P10645 | -1.7969 | 0.2878 | 4.0E-05 | 0.0000 |  |  |  |
| Secretogranin-1 | P05060 | -1.4118 | 0.3758 | 1.7E-03 | 0.0044 |  |  |  |
| Fibrinogen gamma chain | P02679 | -1.3848 | 0.3829 | 1.8E-06 | 0.0000 |  |  |  |
| Dickkopf-related protein 3 | Q9UBP4 | -1.3612 | 0.3893 | 4.7E-05 | 0.0001 |  |  |  |
| Beta-Ala-His dipeptidase | Q96KN2 | -1.3121 | 0.4027 | 1.1E-05 | 0.0000 |  |  |  |
| Superoxide dismutase [Cu-Zn] | P00441 | -1.2616 | 0.4171 | 6.4E-04 | 0.0015 |  |  |  |
| Zinc-alpha-2-glycoprotein | P25311 | -1.2101 | 0.4323 | 4.2E-06 | 0.0000 |  |  |  |
| Alpha-1-acid glycoprotein 2 | P19652 | -1.1496 | 0.4507 | 3.8E-09 | 0.0000 |  |  |  |
| Clusterin | P10909 | -1.1222 | 0.4594 | 5.1E-06 | 0.0000 |  |  |  |
| Major prion protein | P04156 | -1.1142 | 0.4620 | 6.3E-03 | 0.0182 |  |  |  |
| Neurotrimin | Q9P121 | -1.0264 | 0.4909 | 5.5E-04 | 0.0013 |  |  |  |
| Complement C1q subcomponent subunit A | P02745 | -1.0068 | 0.4976 | 7.0E-03 | 0.0194 |  |  |  |
| Neural cell adhesion molecule 1 | P13591 | -1.0033 | 0.4989 | 9.3E-03 | 0.0238 |  |  |  |
| Haptoglobin | P00738 | -0.9502 | 0.5175 | 3.4E-04 | 0.0011 |  |  |  |
| Fibulin-1 | P23142 | -0.9299 | 0.5249 | 2.8E-03 | 0.0081 |  |  |  |
| EGF-containing fibulin-like extracellular matrix protein 1 | Q12805 | -0.9277 | 0.5257 | 6.7E-03 | 0.0191 |  |  |  |
| Alpha-1-acid glycoprotein 1 | P02763 | -0.9193 | 0.5288 | 4.0E-05 | 0.0000 |  |  |  |
| Phospholipid transfer protein | P55058 | -0.9177 | 0.5294 | 5.6E-04 | 0.0013 |  |  |  |
| Plasminogen | P00747 | -0.8758 | 0.5449 | 1.3E-04 | 0.0004 |  |  |  |
| Complement factor H | P08603 | -0.8728 | 0.5461 | 1.1E-04 | 0.0004 |  |  |  |
| Cystatin-C | P01034 | -0.8208 | 0.5661 | 1.9E-02 | 0.0416 |  |  |  |
| Serotransferrin | P02787 | -0.8156 | 0.5682 | 4.4E-10 | 0.0000 |  |  |  |
| Secretogranin-3 | Q8WXD2 | -0.8040 | 0.5728 | 5.4E-03 | 0.0154 |  |  |  |
| Complement factor B | P00751 | -0.8003 | 0.5742 | 6.2E-10 | 0.0000 |  |  |  |
| Serine protease 1 | P07477 | -0.7942 | 0.5767 | 1.6E-07 | 0.0000 |  |  |  |
| Ceruloplasmin | P00450 | -0.7756 | 0.5841 | 8.5E-03 | 0.0225 |  |  |  |
| Immunoglobulin heavy constant alpha 1 | P01876 | -0.7060 | 0.6130 | 8.9E-03 | 0.0230 |  |  |  |
| Angiotensinogen | P01019 | -0.7000 | 0.6156 | 5.6E-06 | 0.0000 |  |  |  |
| Plasma protease C1 inhibitor | P05155 | -0.6906 | 0.6196 | 2.8E-07 | 0.0000 |  |  |  |
| Alpha-2-macroglobulin | P01023 | -0.6352 | 0.6439 | 9.9E-05 | 0.0004 |  |  |  |
| Ectonucleotide pyrophosphatase/phosphodiesterase family member 2 | Q13822 | -0.6197 | 0.6508 | 3.2E-03 | 0.0096 |  |  |  |
| Gelsolin | P06396 | -0.6062 | 0.6569 | 8.4E-04 | 0.0023 |  |  |  |
| Keratin, type I cytoskeletal 10 | P13645 | -0.5870 | 0.6657 | 9.7E-03 | 0.0243 |  |  |  |
| Protein AMBP | P02760 | -0.5808 | 0.6686 | 1.3E-03 | 0.0036 |  |  |  |
| Hemopexin | P02790 | -0.5780 | 0.6699 | 1.1E-09 | 0.0000 |  |  |  |
| Lysosome-associated membrane glycoprotein 2 | P13473 | -0.5394 | 0.6881 | 3.6E-03 | 0.0106 |  |  |  |
| Complement C3 | P01024 | -0.5307 | 0.6922 | 4.9E-05 | 0.0001 |  |  |  |
| Heparin cofactor 2 | P05546 | -0.5015 | 0.7064 | 4.1E-06 | 0.0000 |  |  |  |
| Afamin | P43652 | -0.4822 | 0.7159 | 1.2E-04 | 0.0004 |  |  |  |
| Apolipoprotein A-I | P02647 | -0.4771 | 0.7184 | 4.5E-04 | 0.0013 |  |  |  |
| Prothrombin | P00734 | -0.4521 | 0.7310 | 8.4E-03 | 0.0225 |  |  |  |
| Serum paraoxonase/arylesterase 1 | P27169 | -0.4420 | 0.7361 | 1.7E-02 | 0.0389 |  |  |  |
| Inter-alpha-trypsin inhibitor heavy chain H4 | Q14624 | -0.4250 | 0.7448 | 2.2E-04 | 0.0011 |  |  |  |
| Kininogen-1 | P01042 | -0.4106 | 0.7523 | 6.4E-04 | 0.0015 |  |  |  |
| Apolipoprotein A-IV | P06727 | -0.4094 | 0.7529 | 1.7E-02 | 0.0384 |  |  |  |
| Beta-2-glycoprotein 1 | P02749 | -0.4065 | 0.7544 | 2.8E-04 | 0.0010 |  |  |  |
| Leucine-rich alpha-2-glycoprotein | P02750 | -0.3883 | 0.7641 | 1.7E-02 | 0.0384 |  |  |  |
| Vitamin D-binding protein | P02774 | -0.3735 | 0.7719 | 2.1E-03 | 0.0058 |  |  |  |
| **UniProt Name** | **UniProt ID** | **Log Fold Change** | **Fold Change** | **Student's T-test p-value** | **Student's T-test q-value** |  |  |  |
| Vitronectin | P04004 | -0.3696 | 0.7740 | 1.5E-02 | 0.0372 |  |  |  |
| Albumin | P02768 | 0.1550 | 1.1135 | 4.2E-03 | 0.0122 |  |  |  |
| Ganglioside GM2 activator | P17900 | 0.3212 | 1.2494 | 1.1E-02 | 0.0268 |  |  |  |
| Immunoglobulin kappa variable 2-30 | P06310 | 0.4068 | 1.3257 | 1.5E-02 | 0.0368 |  |  |  |
| Keratin, type II cytoskeletal 1 | P04264 | 0.4463 | 1.3625 | 8.0E-04 | 0.0023 |  |  |  |
| Immunoglobulin heavy variable 3-7 | P01780 | 0.5233 | 1.4373 | 5.6E-03 | 0.0164 |  |  |  |
| Keratin, type II cytoskeletal 5 | P13647 | 0.6198 | 1.5366 | 1.9E-03 | 0.0050 |  |  |  |
| Monocyte differentiation antigen CD14 | P08571 | 0.6203 | 1.5372 | 8.2E-03 | 0.0223 |  |  |  |
| Immunoglobulin kappa variable 1D-16 | P01601 | 0.6534 | 1.5729 | 1.4E-02 | 0.0332 |  |  |  |
| Keratin, type II cytoskeletal 4 | P19013 | 0.6623 | 1.5826 | 5.7E-04 | 0.0013 |  |  |  |
| Lysozyme C | P61626 | 0.7027 | 1.6275 | 1.4E-03 | 0.0037 |  |  |  |
| Immunoglobulin gamma-1 heavy chain | P0DOX5 | 0.7187 | 1.6457 | 1.6E-02 | 0.0365 |  |  |  |
| Immunoglobulin heavy constant gamma 2 | P01859 | 0.7203 | 1.6476 | 4.2E-04 | 0.0011 |  |  |  |
| Keratinocyte proline-rich protein | Q5T749 | 0.7323 | 1.6613 | 9.3E-03 | 0.0234 |  |  |  |
| Immunoglobulin lambda variable 3-27 | P01718 | 0.7466 | 1.6778 | 1.0E-02 | 0.0253 |  |  |  |
| Immunoglobulin heavy variable 3-43D | P0DP04 | 0.7909 | 1.7302 | 1.3E-02 | 0.0335 |  |  |  |
| Immunoglobulin heavy variable 4-31 | P0DP07 | 0.8060 | 1.7483 | 2.1E-03 | 0.0057 |  |  |  |
| Inter-alpha-trypsin inhibitor heavy chain H2 | P19823 | 0.8199 | 1.7653 | 1.7E-02 | 0.0383 |  |  |  |
| Junction plakoglobin | P14923 | 0.8451 | 1.7964 | 2.2E-02 | 0.0491 |  |  |  |
| Insulin-like growth factor-binding protein 4 | P22692 | 0.8455 | 1.7969 | 2.3E-03 | 0.0066 |  |  |  |
| Immunoglobulin kappa light chain | P0DOX7 | 0.8834 | 1.8447 | 3.8E-05 | 0.0000 |  |  |  |
| Protein S100-A9 | P06702 | 0.9609 | 1.9465 | 9.3E-03 | 0.0234 |  |  |  |
| Immunoglobulin kappa variable 1-33 | P01594 | 0.9744 | 1.9648 | 7.5E-03 | 0.0203 |  |  |  |
| Immunoglobulin kappa variable 3-20 | P01619 | 0.9831 | 1.9767 | 7.0E-09 | 0.0000 |  |  |  |
| Immunoglobulin kappa variable 4-1 | P06312 | 1.0849 | 2.1213 | 2.5E-06 | 0.0000 |  |  |  |
| Apolipoprotein A-II | P02652 | 1.1315 | 2.1909 | 2.0E-08 | 0.0000 |  |  |  |
| Immunoglobulin kappa variable 3-11 | P04433 | 1.3222 | 2.5005 | 8.5E-08 | 0.0000 |  |  |  |
| Hemoglobin subunit beta | P68871 | 1.3334 | 2.5199 | 2.2E-03 | 0.0060 |  |  |  |
| Biotinidase | P43251 | 1.3410 | 2.5333 | 4.7E-06 | 0.0000 |  |  |  |
| Keratin, type I cytoskeletal 17 | Q04695 | 1.4629 | 2.7567 | 8.6E-06 | 0.0000 |  |  |  |
| Submaxillary gland androgen-regulated protein 3B | P02814 | 1.5152 | 2.8583 | 1.5E-07 | 0.0000 |  |  |  |
| Transthyretin | P02766 | 1.6588 | 3.1574 | 3.5E-09 | 0.0000 |  |  |  |
| Immunoglobulin kappa variable 1-27 | A0A075B6S5 | 2.0059 | 4.0164 | 4.8E-05 | 0.0001 |  |  |  |
| Immunoglobulin heavy variable 1-18 | A0A0C4DH31 | 2.1898 | 4.5623 | 6.0E-07 | 0.0000 |  |  |  |

**Additional table 1.** Analysis of SWATH-MS data comparing non-MS to MS CSF samples. Only proteins with statistically significant differences are shown. A color-coded legend highlights upregulation and downregulation in the log2FC column
