# Supplementary material for: EBI2-oxysterol signalling regulates VE-cadherin expression and multiple sclerosis CD4+ T cell attachment to a human tri-cell spheroid blood-brain barrier model
Source: Brain Behav Immun Health. 2025 Jun 20;47:101045. doi: 10.1016/j.bbih.2025.101045 (PMC12246718; doi:10.1016/j.bbih.2025.101045)
Supplement: Multimedia component 2 [file mmc2.docx]

| **Group** | **Gender** | **Age** | **Samples used for:** |
| --- | --- | --- | --- |
| MS | M | 34 | ELISA (CSF); proteomics |
| MS | F | 24 | ELISA (CSF, serum); in vitro experiments; proteomics |
| MS | F | 27 | in vitro experiments; proteomics |
| MS | F | 22 | ELISA (CSF, serum, T cells); in vitro experiments and adhesion assay; proteomics |
| MS | F | 34 | ELISA (T cells); adhesion assay |
| MS | F | 25 | ELISA (CSF, serum); in vitro experiments; proteomics |
| MS | M | 25 | ELISA (CSF, serum, T cells); in vitro experiments and adhesion assay; proteomics |
| MS | F | 28 | in vitro experiments; proteomics |
| MS | F | 25 | ELISA (CSF, T cells); in vitro experiments and adhesion assay; proteomics |
| MS | F | 19 | ELISA (CSF, serum, T cells); adhesion assay; proteomics |
| MS | M | 35 | ELISA (CSF, T cells); adhesion assay; proteomics |
| MS | M | 37 | ELISA (CSF, serum); adhesion assay; proteomics |
| MS | F | 25 | ELISA (CSF, serum); proteomics |
| MS | M | 33 | in vitro experiments; proteomics |
| MS | M | 28 | ELISA (CSF, serum); proteomics |
| MS | M | 28 | ELISA (CSF, serum); proteomics |
| MS | F | 43 | ELISA (CSF, serum); proteomics |
| Non-MS | F | 57 | ELISA (CSF, serum); in vitro experiments |
| Non-MS | F | 36 | ELISA (serum) |
| Non-MS | F | 29 | ELISA (serum) |
| Non-MS | F | 57 | ELISA (CSF, serum); in vitro experiments |
| Non-MS | F | 64 | ELISA (CSF); in vitro experiments; proteomics |
| Non-MS | F | 23 | ELISA (CSF, serum); in vitro experiments and adhesion assay |
| Non-MS | M | 40 | ELISA (CSF, serum. T cells); in vitro experiments and adhesion assay |
| Non-MS | F | 34 | ELISA (CSF, serum, T cells); adhesion assay; proteomics |
| Non-MS | F | 25 | ELISA (serum) |
| Non-MS | M | 23 | ELISA (serum) |
| Non-MS | M | 30 | ELISA (CSF, serum, T cells); in vitro experiments and adhesion assay; proteomics |
| Non-MS | F | 39 | ELISA (T cells); adhesion assay; proteomics |
| Non-MS | F | 37 | ELISA (T cells); adhesion assay; proteomics |
| Non-MS | F | 40 | ELISA (T cells); adhesion assay; proteomics |

**Additional table 2.** Detailed patient information and group and in vitro experiment allocation
