# Supplementary material for: EBI2-oxysterol signalling regulates VE-cadherin expression and multiple sclerosis CD4+ T cell attachment to a human tri-cell spheroid blood-brain barrier model
Source: Brain Behav Immun Health. 2025 Jun 20;47:101045. doi: 10.1016/j.bbih.2025.101045 (PMC12246718; doi:10.1016/j.bbih.2025.101045)
Supplement: Multimedia component 3 [file mmc3.docx]

| **Group** | **Age-at-death** | **Gender** | **Brain region** | **Lesion status** | **Post-mortem delay** | **Samples used for:** |
| --- | --- | --- | --- | --- | --- | --- |
| MS | 50 | F | periventricular | Chronic inactive | unknown | microvessels; mRNA, ICC, WB |
| MS | 87 | F | periventricular | Chronic inactive | unknown | microvessels; mRNA, ICC |
| MS | 86 | F | periventricular | Unknown | unknown | microvessels; mRNA, ICC |
| MS | 41 | F | periventricular | Chronic inactive | 3 h | microvessels; mRNA |
| MS | 53 | F | periventricular | Chronic inactive | unknown | microvessels; mRNA |
| MS | 69 | F | centrum semiovale | Chronic inactive with focal chronic activity | unknown | microvessels; mRNA, WB |
| Non-MS | 68 | M | periventricular | N/A | 5 h | microvessels; mRNA |
| Non-MS | 22 | M | periventricular | N/A | 18.5 h | microvessels; mRNA |
| Non-MS | 84 | M | periventricular | N/A | 4.5 h | microvessels; mRNA, ICC, WB |
| Non-MS | 56 | F | periventricular | N/A | 2 h | microvessels; mRNA |
| Non-MS | 80 | M | periventricular | N/A | unknown | microvessels; mRNA, ICC, WB |

**Additional table 3.** Detailed patient information for the frozen brain samples
