# Supplementary material for: EBI2-oxysterol signalling regulates VE-cadherin expression and multiple sclerosis CD4+ T cell attachment to a human tri-cell spheroid blood-brain barrier model
Source: Brain Behav Immun Health. 2025 Jun 20;47:101045. doi: 10.1016/j.bbih.2025.101045 (PMC12246718; doi:10.1016/j.bbih.2025.101045)
Supplement: Multimedia component 4 [file mmc4.pptx]

## Slide 1
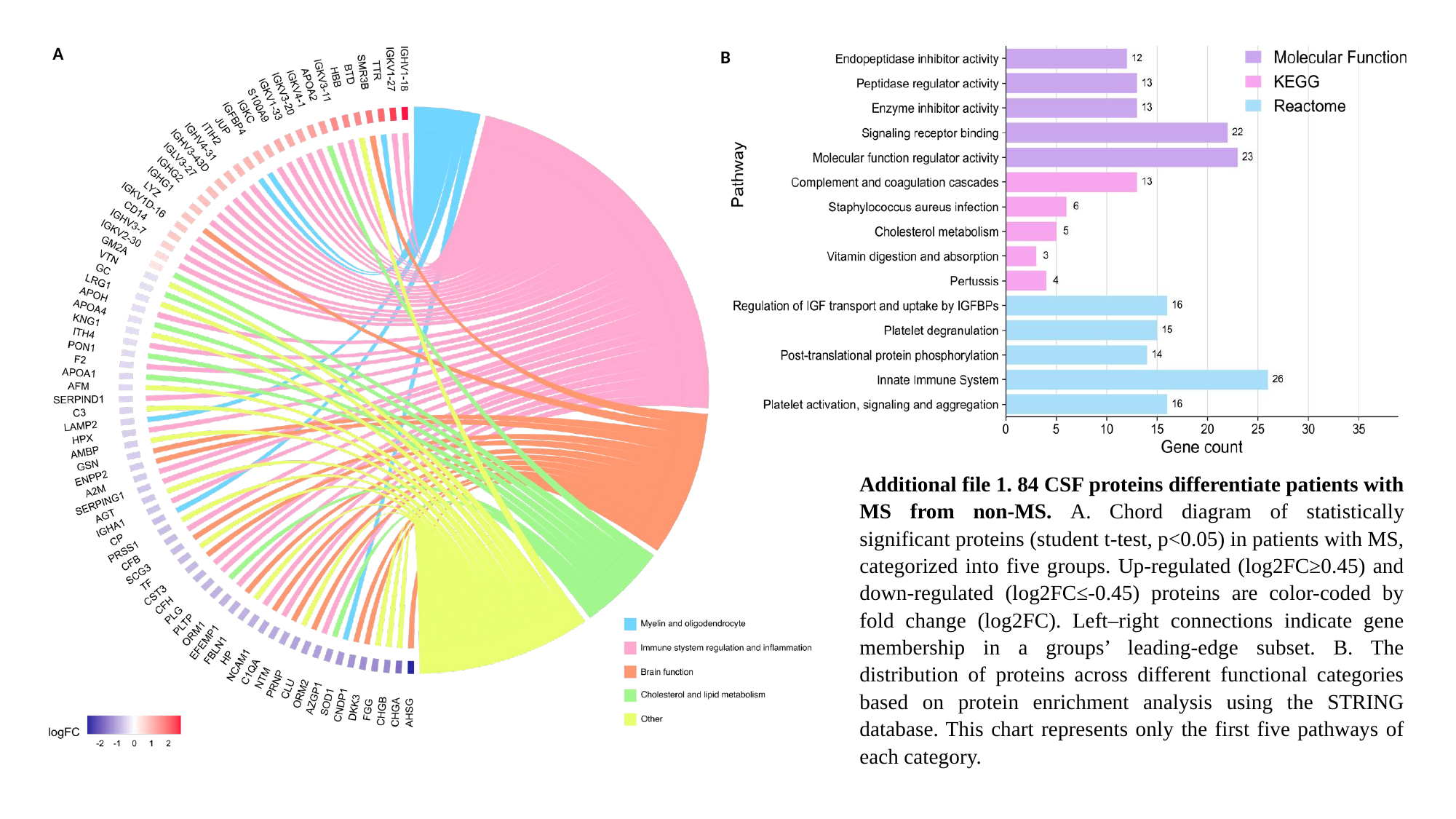

A
B
Additional file 1. 84 CSF proteins differentiate patients with MS from non-MS. A. Chord diagram of statistically significant proteins (student t-test, p<0.05) in patients with MS, categorized into five groups. Up-regulated (log2FC≥0.45) and down-regulated (log2FC≤-0.45) proteins are color-coded by fold change (log2FC). Left–right connections indicate gene membership in a groups’ leading-edge subset. B. The distribution of proteins across different functional categories based on protein enrichment analysis using the STRING database. This chart represents only the first five pathways of each category.

## Slide 2
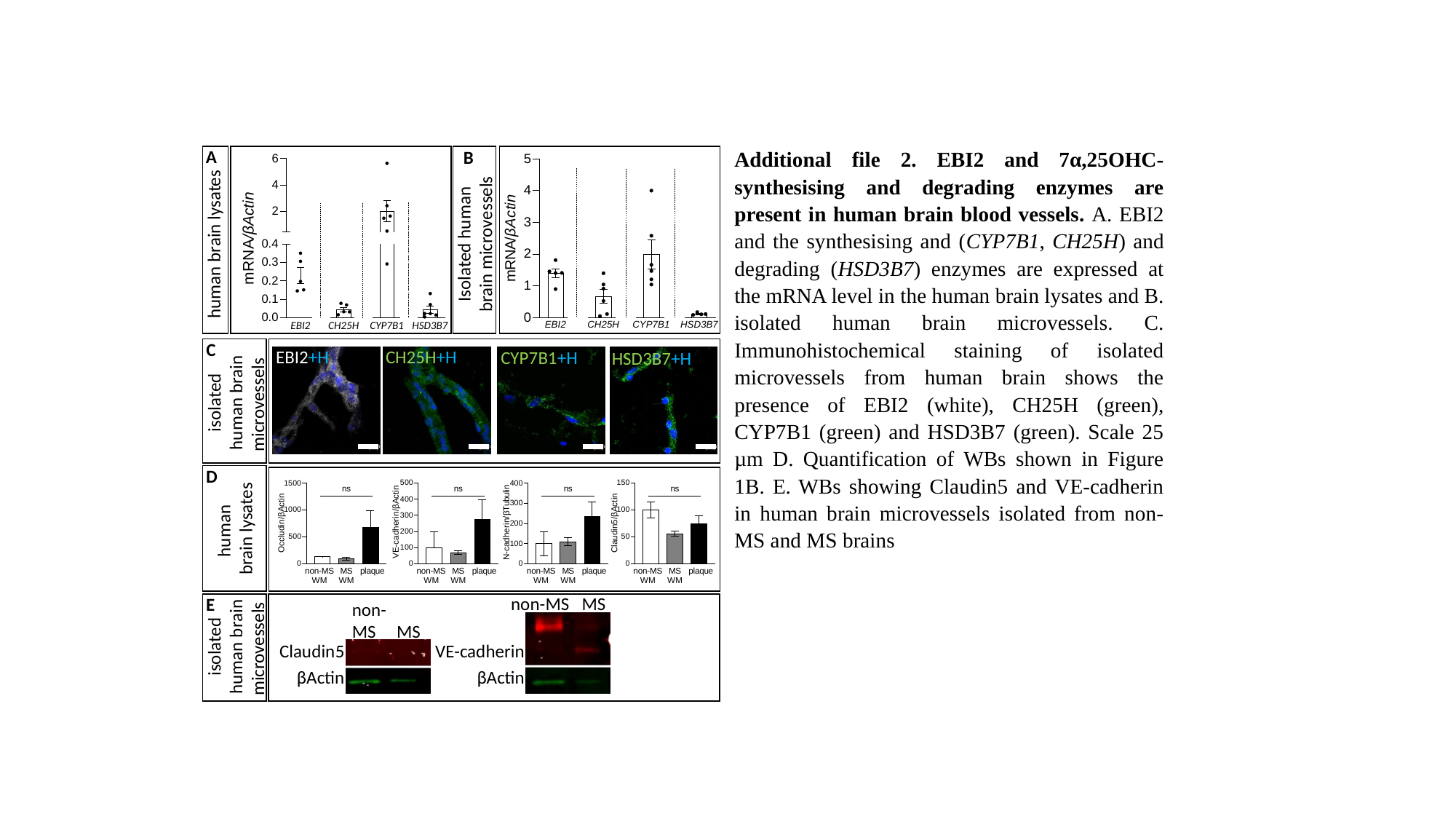

Additional file 2. EBI2 and 7α,25OHC-synthesising and degrading enzymes are present in human brain blood vessels. A. EBI2 and the synthesising and (CYP7B1, CH25H) and degrading (HSD3B7) enzymes are expressed at the mRNA level in the human brain lysates and B. isolated human brain microvessels. C. Immunohistochemical staining of isolated microvessels from human brain shows the presence of EBI2 (white), CH25H (green), CYP7B1 (green) and HSD3B7 (green). Scale 25 µm D. Quantification of WBs shown in Figure 1B. E. WBs showing Claudin5 and VE-cadherin in human brain microvessels isolated from non-MS and MS brains
A
B
Isolated human brain microvessels
human brain lysates
C
EBI2+H
CH25H+H
CYP7B1+H
HSD3B7+H
isolated human brain microvessels
D
human
brain lysates
non-MS MS
E
non-
MS MS
isolated human brain microvessels
Claudin5
VE-cadherin
βActin
βActin

## Slide 3
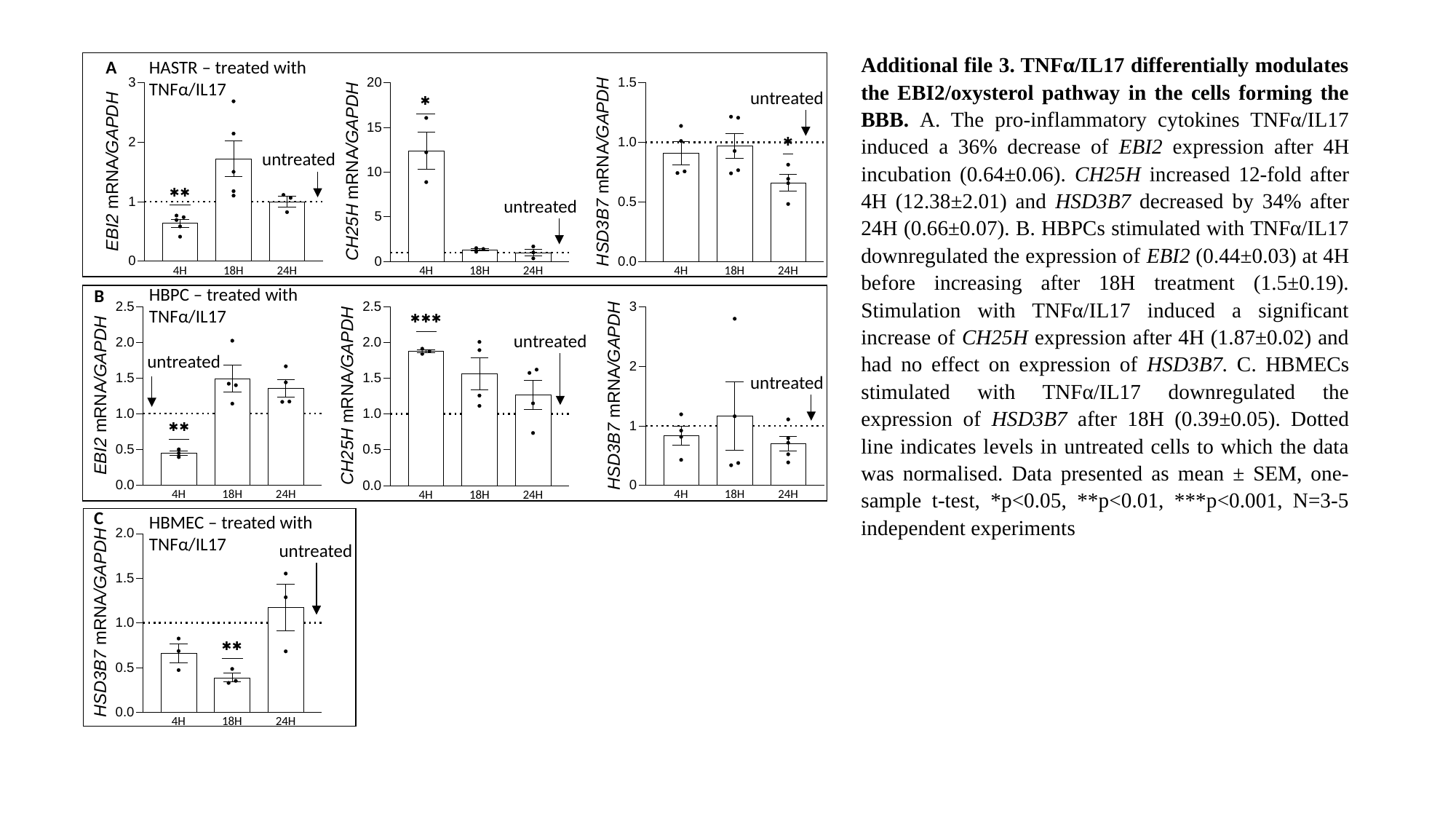

Additional file 3. TNFα/IL17 differentially modulates the EBI2/oxysterol pathway in the cells forming the BBB. A. The pro-inflammatory cytokines TNFα/IL17 induced a 36% decrease of EBI2 expression after 4H incubation (0.64±0.06). CH25H increased 12-fold after 4H (12.38±2.01) and HSD3B7 decreased by 34% after 24H (0.66±0.07). B. HBPCs stimulated with TNFα/IL17 downregulated the expression of EBI2 (0.44±0.03) at 4H before increasing after 18H treatment (1.5±0.19). Stimulation with TNFα/IL17 induced a significant increase of CH25H expression after 4H (1.87±0.02) and had no effect on expression of HSD3B7. C. HBMECs stimulated with TNFα/IL17 downregulated the expression of HSD3B7 after 18H (0.39±0.05). Dotted line indicates levels in untreated cells to which the data was normalised. Data presented as mean ± SEM, one-sample t-test, *p<0.05, **p<0.01, ***p<0.001, N=3-5 independent experiments
HASTR – treated with TNFα/IL17
A
untreated
untreated
untreated
HBPC – treated with TNFα/IL17
B
untreated
untreated
untreated
C
HBMEC – treated with TNFα/IL17
untreated

## Slide 4
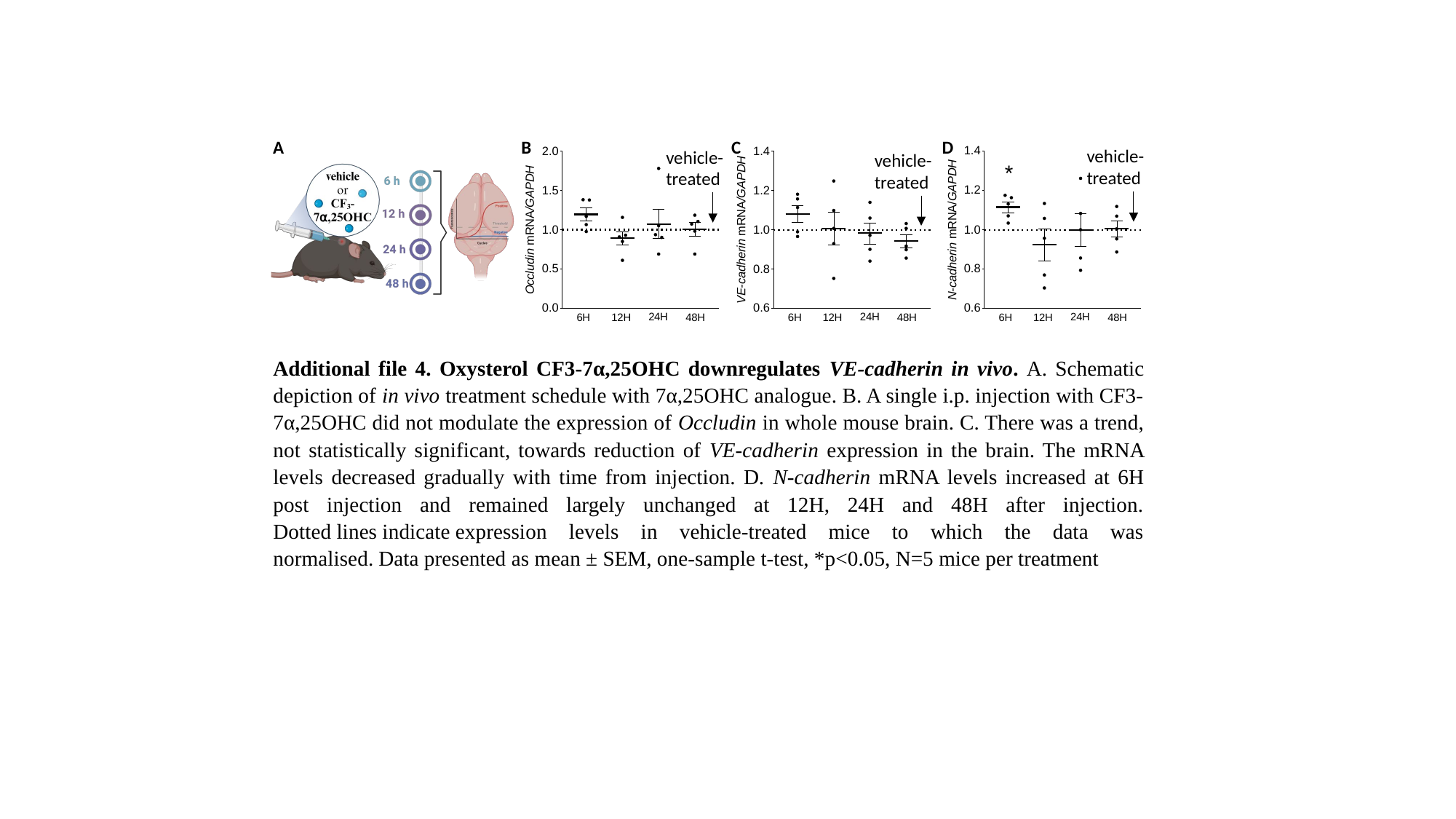

A
B
C
D
vehicle-treated
vehicle-treated
vehicle-treated
Additional file 4. Oxysterol CF3-7α,25OHC downregulates VE-cadherin in vivo. A. Schematic depiction of in vivo treatment schedule with 7α,25OHC analogue. B. A single i.p. injection with CF3-7α,25OHC did not modulate the expression of Occludin in whole mouse brain. C. There was a trend, not statistically significant, towards reduction of VE-cadherin expression in the brain. The mRNA levels decreased gradually with time from injection. D. N-cadherin mRNA levels increased at 6H post injection and remained largely unchanged at 12H, 24H and 48H after injection. Dotted lines indicate expression levels in vehicle-treated mice to which the data was normalised. Data presented as mean ± SEM, one-sample t-test, *p<0.05, N=5 mice per treatment

## Slide 5
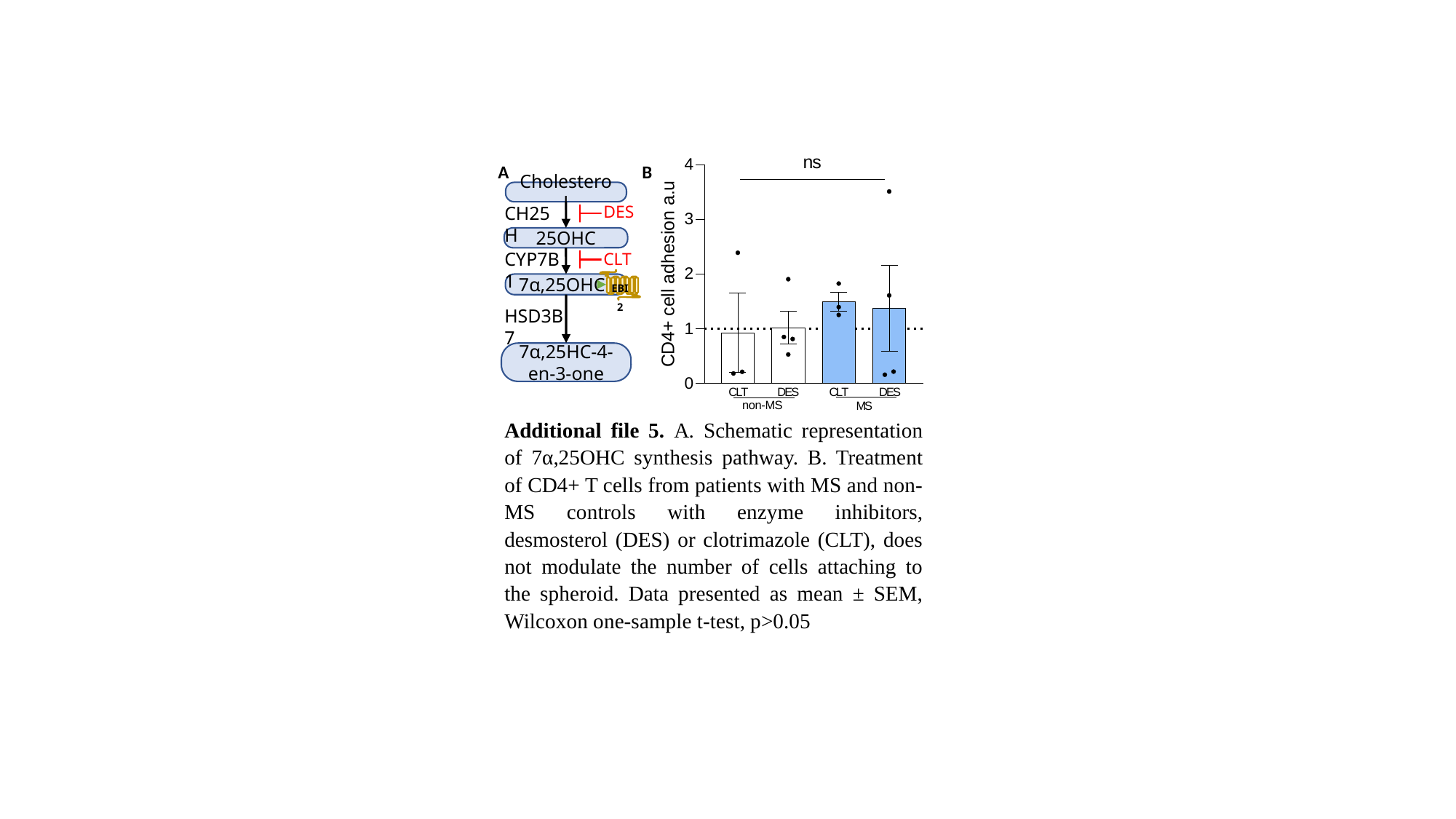

A
B
Cholesterol
DES
CH25H
25OHC
CYP7B1
CLT
EBI2
7α,25OHC
HSD3B7
7α,25HC-4-en-3-one
Additional file 5. A. Schematic representation of 7α,25OHC synthesis pathway. B. Treatment of CD4+ T cells from patients with MS and non-MS controls with enzyme inhibitors, desmosterol (DES) or clotrimazole (CLT), does not modulate the number of cells attaching to the spheroid. Data presented as mean ± SEM, Wilcoxon one-sample t-test, p>0.05

## Slide 6
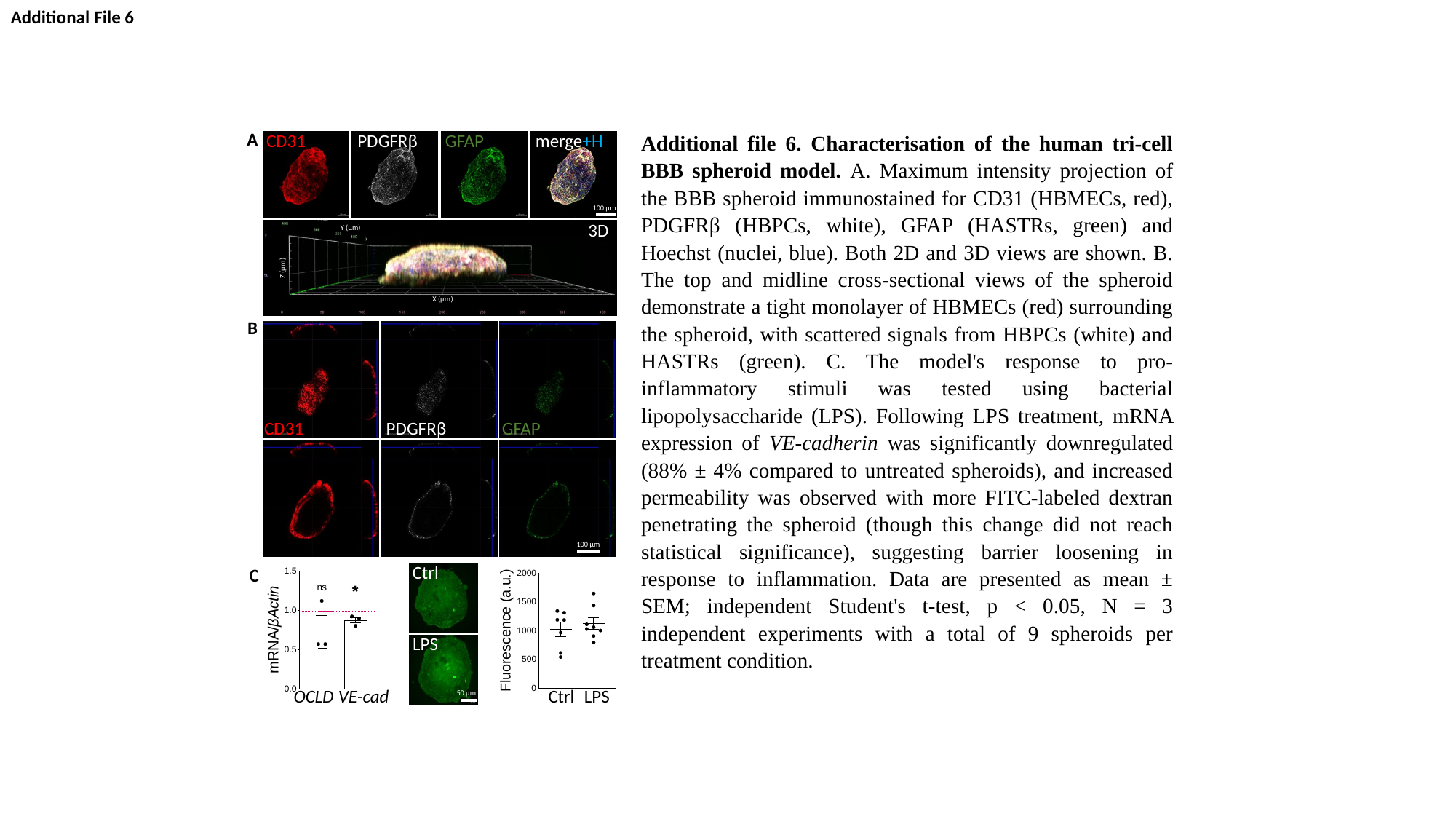

Additional File 6
A
CD31
PDGFRβ
GFAP
merge+H
100 µm
Y (µm)
3D
Y (µm)
Z (µm)
X (µm)
B
100 µm
CD31
PDGFRβ
GFAP
Additional file 6. Characterisation of the human tri-cell BBB spheroid model. A. Maximum intensity projection of the BBB spheroid immunostained for CD31 (HBMECs, red), PDGFRβ (HBPCs, white), GFAP (HASTRs, green) and Hoechst (nuclei, blue). Both 2D and 3D views are shown. B. The top and midline cross-sectional views of the spheroid demonstrate a tight monolayer of HBMECs (red) surrounding the spheroid, with scattered signals from HBPCs (white) and HASTRs (green). C. The model's response to pro-inflammatory stimuli was tested using bacterial lipopolysaccharide (LPS). Following LPS treatment, mRNA expression of VE-cadherin was significantly downregulated (88% ± 4% compared to untreated spheroids), and increased permeability was observed with more FITC-labeled dextran penetrating the spheroid (though this change did not reach statistical significance), suggesting barrier loosening in response to inflammation. Data are presented as mean ± SEM; independent Student's t-test, p < 0.05, N = 3 independent experiments with a total of 9 spheroids per treatment condition.
Ctrl
C
LPS
OCLD
VE-cad
Ctrl
LPS
50 µm
